# The association between the tumor immune microenvironments and clinical outcome in low‐grade, early‐stage endometrial cancer patients

**DOI:** 10.1002/path.6012

**Published:** 2022-10-25

**Authors:** Álvaro López‐Janeiro, María Villalba‐Esparza, María Emilia Brizzi, Daniel Jiménez‐Sánchez, Ignacio Ruz‐Caracuel, Ece Kadioglu, Ivan Masetto, Virginie Goubert, David Garcia‐Ros, Ignacio Melero, Alberto Peláez‐García, David Hardisson, Carlos E de Andrea

**Affiliations:** ^1^ Department of Pathology Hospital Universitario La Paz, IdiPAZ Madrid Spain; ^2^ Department of Pathology, Clínica Universidad de Navarra University of Navarra Pamplona Spain; ^3^ Center for Biomedical Research in the Cancer Network (Centro de Investigación Biomédica en Red de Cáncer, CIBERONC) Instituto de Salud Carlos III Madrid Spain; ^4^ Department of Pathology Hospital Universitario Ramón y Cajal, IRYCIS Madrid Spain; ^5^ Lunaphore Technologies SA Tolochenaz Switzerland; ^6^ Akoya Biosciences Marlborough MA USA; ^7^ Department of Immunology and Immunotherapy Clínica Universidad de Navarra Pamplona Spain; ^8^ Program of Immunology and Immunotherapy CIMA Universidad de Navarra Pamplona Spain; ^9^ Molecular Pathology and Therapeutic Targets Group La Paz University Hospital (IdiPAZ) Madrid Spain; ^10^ Faculty of Medicine Universidad Autónoma de Madrid Madrid Spain; ^11^ Present address: Department of Pathology, Clínica Universidad de Navarra University of Navarra Pamplona Spain

**Keywords:** endometrial cancer, low grade, prognosis, immune microenvironment, multiplex quantitative immunofluorescence, clinical outcome

## Abstract

Endometrial tumors show substantial heterogeneity in their immune microenvironment. This heterogeneity could be used to improve the accuracy of current outcome prediction tools. We assessed the immune microenvironment of 235 patients diagnosed with low‐grade, early‐stage endometrial cancer. Multiplex quantitative immunofluorescence was carried out to measure CD8, CD68, FOXP3, PD‐1, and PD‐L1 markers, as well as cytokeratin (CK), on tissue microarrays. Clustering results revealed five robust immune response patterns, each associated with specific immune populations, cell phenotypes, and cell spatial clustering. Most samples (69%) belonged to the immune‐desert subtype, characterized by low immune cell densities. Tumor‐infiltrating lymphocyte (TIL)‐rich samples (4%) displayed high CD8^+^ T‐cell infiltration, as well as a high percentage of CD8/PD‐1^+^ cells. Immune‐exclusion samples (19%) displayed the lowest CD8^+^ infiltration combined with high PD‐L1 expression levels in CK^+^ tumor cells. In addition, they demonstrated high tumor cell spatial clustering as well as increased spatial proximity of CD8^+^/PD‐1^+^ and CK/PD‐L1^+^ cells. FOXP3 and macrophage‐rich phenotypes (3% and 4% of total samples) displayed relatively high levels of FOXP3^+^ regulatory T‐cells and CD68^+^ macrophages, respectively. These phenotypes correlated with clinical outcomes, with immune‐exclusion tumors showing an association with tumor relapse. When compared with prediction models built using routine pathological variables, models optimized with immune variables showed increased outcome prediction capacity (AUC = 0.89 versus 0.78) and stratification potential. The improved prediction capacity was independent of mismatch repair protein status and adjuvant radiotherapy treatment. Further, immunofluorescence results could be partially recapitulated using single‐marker immunohistochemistry (IHC) performed on whole tissue sections. TIL‐rich tumors demonstrated increased CD8^+^ T‐cells by IHC, while immune‐exclusion tumors displayed a lack of CD8^+^ T‐cells and frequent expression of PD‐L1 in tumor cells. Our results demonstrate the capability of the immune microenvironment to improve standard prediction tools in low‐grade, early‐stage endometrial carcinomas. © 2022 The Authors. *The Journal of Pathology* published by John Wiley & Sons Ltd on behalf of The Pathological Society of Great Britain and Ireland.

## Introduction

Endometrial cancer is the most common gynecologic malignancy in developed countries, with the majority of cases diagnosed as low‐grade, early‐stage disease with good clinical outcome [1]. Currently, the management of low‐risk, early‐stage disease (FIGO stage I–II, G1–G2) is surgery, followed by adjuvant radio‐ or brachy‐therapy for patients with high‐risk features [2]. However, 5–10% of these patients will suffer recurrence [3]. Therefore, a better understanding of recurrence development is needed to guide therapeutic management.

Although molecular subtypes have demonstrated an association with clinical outcome [4, 5], their translation into routine practice through risk stratification of newly diagnosed patients needs to be further determined [6, 7, 8]. Low‐grade, early‐stage endometrial carcinomas are associated with mismatch repair protein deficiency and a low number of somatic copy‐number alterations (endometrioid‐like). Both molecular subtypes have shown a weaker association with prognosis compared with *POLE*‐mutated and p53mut/copy‐number high (serous‐like) molecular subtypes [4, 5]. Other novel molecular features such as the presence of beta‐catenin mutations appear to involve a worse prognosis in this specific subset of patients [9] but have still not gained wide acceptance in routine clinical practice.

The tumor immune microenvironment (TIME) is closely associated with tumor biology. The spatial immune infiltration patterns in the tumor core are traditionally classified into immune‐desert, immune‐exclusion, and inflamed tumors [10]. This classification has been used to improve the prediction ability of classical tumor staging in colorectal cancer [11, 12].

Endometrial cancer has shown a heterogeneous immune infiltrate, varying between tumor grades, and molecular subtypes [13, 14]. We have previously identified that tumor‐specific immune responses were dysregulated in low‐grade, early‐stage tumors [15]. Thus, the upregulation of immune‐related genes and increased immune cell infiltration could be used to improve current prediction tools.

Here, we explored the heterogeneous TIME in a large and well‐characterized cohort of patients with low‐grade, early‐stage endometrial carcinoma, and assessed the association between the complex tumor‐immune interrelations and the risk of recurrence compared with standard clinicopathological features.

## Materials and methods

### Patient selection and tissue microarray (TMA) construction

We retrospectively recruited consecutive patients with endometrial carcinoma from January 2003 to December 2015. Patients included met the following criteria: (1) surgical treatment was performed in a single institution (University Hospital La Paz, Madrid, Spain); (2) using the 2009 International Federation of Gynecology and Obstetrics (FIGO) classification, all tumors were at stages I and II and were low‐grade (G1 or G2) endometrioid carcinomas; (3) all tumors had a wild‐type expression pattern of p53 protein by immunohistochemistry; and (4) patients did not receive neoadjuvant/adjuvant systemic treatment or immunotherapy. Pathologic re‐evaluation was carried out by a pathologist who selected two random areas with viable tumor for TMA construction. Selected areas contained variable proportions of tumor and stromal tissue. The infiltration border was not considered. Two 1.2‐mm cores were taken from each formalin‐fixed, paraffin‐embedded (FFPE) tissue block. All patients included in the analysis signed a written informed consent. The present study was conducted in accordance with the Helsinki Declaration and was approved by the local Ethics Committee (protocol code HULP: PI‐3108, 28 February 2018).

### Multiplex immunofluorescence

The validation pipeline and details of the development of the multiplex immunolabeling protocol have been described previously by our group and are shown in supplementary material, Figure S1 and Supplementary materials and methods [15, 16]. Multiplex immunofluorescence staining was performed using the LabSat® Research platform (Lunaphore Technologies, Tolochenaz, Switzerland), a fully automated tissue‐staining instrument for rapid immunostaining which utilizes a microfluidic technology for the rapid and uniform delivery of reagents to tissue samples [17]. In brief, each TMA section was subjected to six successive, automated rounds of antibody staining, including pan‐cytokeratin, CD8, CD68, FOXP3, PD‐1, and PD‐L1. Nuclei were counterstained with spectral DAPI (Akoya Biosciences, Marlborough, MA, USA). A full protocol is provided in Supplementary materials and methods. TMAs were scanned using a PhenoImager HT Automated Quantitative Pathology Imaging System (Akoya Biosciences). Image analysis was performed using the inForm software framework (version 2.4.8, Akoya Biosciences), shown in Supplementary materials and methods.

### Spatial immune infiltration patterns and their correlation with clinical outcome

To identify immune profiles, we performed hierarchical clustering using the immune population densities (see Supplementary materials and methods and supplementary material, Table S1).

The correlation between immune phenotypes and clinical outcomes was assessed by the presence or absence of each of the immune clusters, FIGO stage, tumor grade, presence of lymphovascular space invasion (LVSI), and adjuvant radiotherapy. Given that most tumors recur in the first 3 years after diagnosis [3], we excluded patients with loss of follow‐up within 36 months after diagnosis. Follow‐up data regarding tumor relapse were obtained from the electronic clinical records. Patient follow‐up was considered complete after 10 years (supplementary material, Table S2).

To assess the tumor relapse prediction power, we fitted a logistic regression model using key parameters for risk stratification according to current guidelines which includes FIGO stage, tumor grade, and LVSI [18]. This model was used as a reference. Next, we optimized the reference prediction model using the immune profiles. Feature selection was done through an iterative process of model fitting and cross‐validation using the mlr R package [19]. Every single combination of the clinicopathological variables and the immune phenotype variables was used to fit logistic regression models. Each model was subsequently cross‐validated using a two‐fold 20‐times cross‐validation strategy. The model with the lowest mean misclassification error was selected. The predictive power of the reference and optimized models was calculated using the area under the curve (AUC). The *pROC* package was used to calculate AUCs and the Youden index to select the best performing cut‐off values [20]. To check if model outperformance was dependent on sampling error, we assessed the prediction performance of both models over 10,000 bootstrapped samples.

### Mismatch repair protein (MMRP) status and *POLE* and *CTNNB1* mutation detection

MMRP status and p53 expression pattern were determined as described previously [21, 22] (see Supplementary materials and methods). Detection of mutations in the *POLE* exonuclease domain (exons 9, 11, 13, and 14) and *CTNNB1* exon 3 were analyzed by Sanger sequencing as described previously [23].

### Immunohistochemical assays of whole‐slide tumor sections

Validation studies of the immunofluorescence results were performed on whole‐slide tumor tissue. Since the amount of stroma present in the TMA cores was variable, only intra‐tumoral markers were quantified. A tree model using the *rpart* R package was used [24] to screen the intra‐tumoral immune markers measured by immunofluorescence. We arbitrarily selected 20 representative cases for IHC validation. We prioritized patients with tissue blocks with abundant tumor that provided two evaluable tumor cores for TMA construction. In addition, tumors demonstrating the same immune cluster across the two cores were selected if possible. Cases belonging to all phenotypes were included in the IHC validation. Given their strong association with prognosis, samples from immune‐exclusion phenotypes were overrepresented (7 out of 20 cases). Consecutive sections from tissue blocks were stained for CD8, CD68, FOXP3, PD‐L1, and hematoxylin/eosin (see Supplementary materials and methods) and scored by a pathologist blinded to the identity of the tumor immune phenotype.

### Statistical analysis

All analyses were carried out using the R (4.0.1) environment [25] (see Supplementary materials and methods). The *P* values are represented as follows: *p < 0.05, **p < 0.01, ***p < 0.001, ****p < 0.0001.

## Results

### Low‐grade, early‐stage endometrial carcinomas show diverse immune phenotypes

In total, 404 TMA cores were analyzed from the tumor blocks of 235 patients. For 169 of these tumor blocks, two tumor cores were analyzable, while a single core per tumor sample was found to be adequate for the remaining 66 patients.

**Figure 1:**
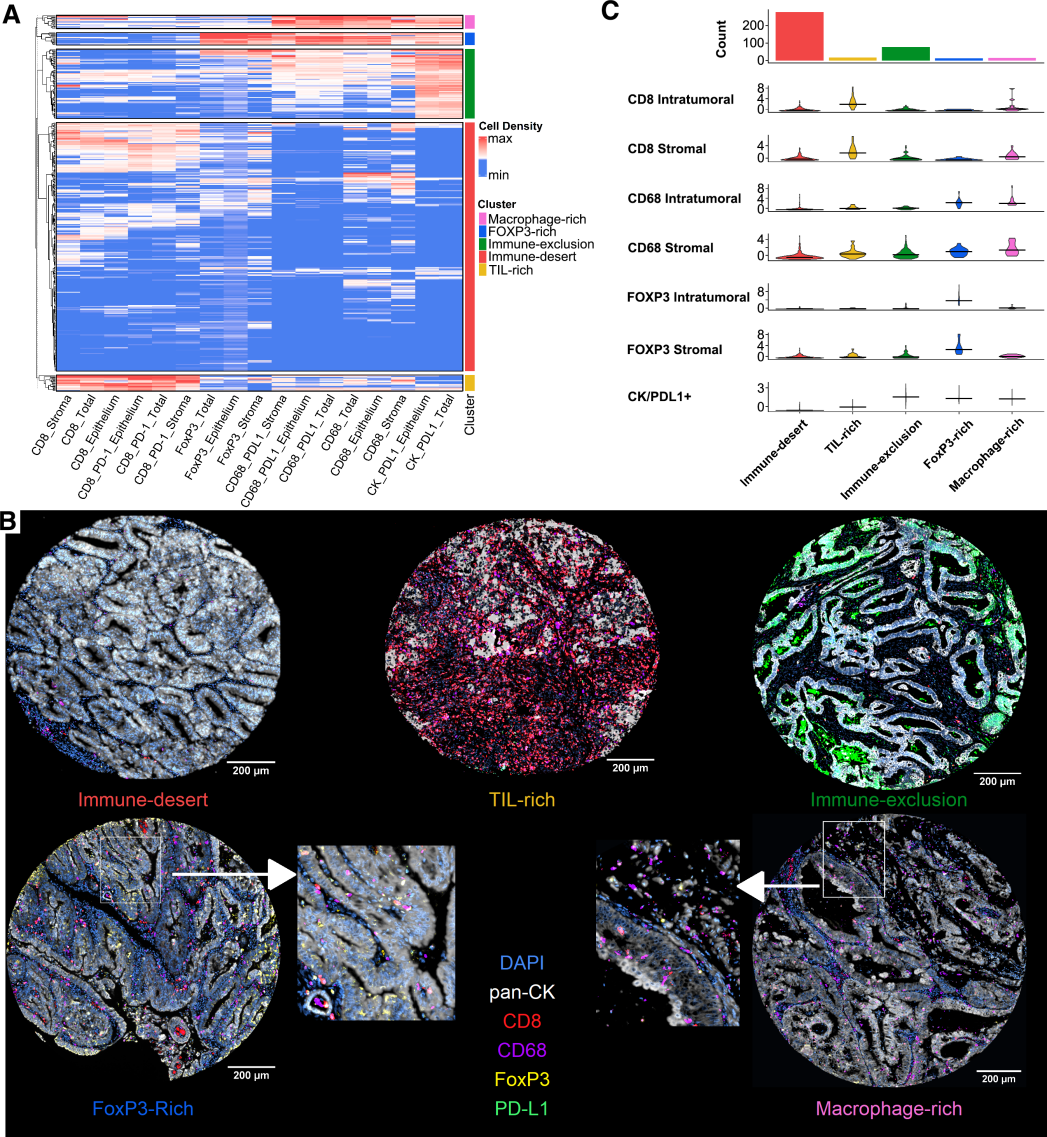
Clustering results. (A) Heatmap representing the cell densities of immune variables across samples. The side color bar represents immune clusters. (B) Representative multiplex immunofluorescence images from each immune phenotype. (C) Violin plot representing selected immune variable distribution (cell density measured as cells/mm^2^) across immune clusters. The median is shown as a cross‐bar. The variables shown are scaled.

Correlation between the densities of all immune cell phenotypes is shown in supplementary material, Figure S2. Unsupervised hierarchical clustering analysis identified five robust clusters (Figure 1A,B). Cell density distribution across clusters is depicted in Figure 1C. Cluster 1 was the most abundant cluster type, representing 279 out of the 404 analyzed TMA cores (69.1%). It was characterized by an overall low immune cell density infiltrate and was named immune‐desert. Immune‐desert samples demonstrated the lowest infiltration density of tumor‐associated macrophages (TAMs) [overall median density across all compartments (OMD) = 134.32 CD68^+^ cells/mm^2^] and the second lowest infiltration density of CD8^+^ T‐cells (OMD = 109.63 CD8^+^ T‐cells/mm^2^). In sharp contrast, cluster 2 showed high numbers of tumor‐infiltrating CD8 T‐lymphocytes (OMD = 695.45 CD8^+^ T‐cells/mm^2^) and was named TIL‐rich. Cluster 3 was characterized by the lowest CD8^+^ cell infiltration (OMD = 106.87 CD8^+^ T‐cells/mm^2^) and the highest density of CK^+^/PD‐L1^+^ cells (OMD = 6,445.38 cells/mm^2^) and was named immune‐exclusion. Finally, clusters 4 and 5 were characterized by relatively high FOXP3^+^ regulatory T‐cell infiltration (FOXP3‐rich) and CD68^+^ macrophages (macrophage‐rich), respectively. The immune‐exclusion cluster represented 78 (19.3%) out of the 404 total cores, while 18 (4.5%), 14 (3.5%), and 15 (3.7%) belonged to less frequent TIL‐rich, FOXP3‐rich, and macrophage‐rich clusters, respectively. Furthermore, the Jaccard indices demonstrated fair cluster robustness, with most clusters showing similarity coefficients above 0.5 over 100 bootstrapped samples.

MMRP expression, *POLE* mutation status, and *CTNNB1* mutation status were available for 89.1% (360/404), 67.1% (271/404), and 83.4% (337/404) of the samples, respectively. Loss of MMRP expression was relatively homogeneous across clusters. However, a slight enrichment of MMRP‐deficient cases in TIL‐rich and immune‐exclusion was noted for the 75 samples belonging to MMRP‐deficient tumors (Fisher exact test *p* < 0.001). Twelve cores belonged to *POLE*‐mutated tumors and were enriched in TIL‐rich and FOXP3‐rich phenotypes, although not significantly (Fisher exact test *p* = 0.08). Finally, the distribution of the 31 samples belonging to *CTNNB1*‐mutated tumors showed a slight non‐significant enrichment in immune‐exclusion samples (Fisher exact test *p* = 0.29) (supplementary material, Figure S2B).

### Cell phenotypes vary across the immune clusters

**Figure 2:**
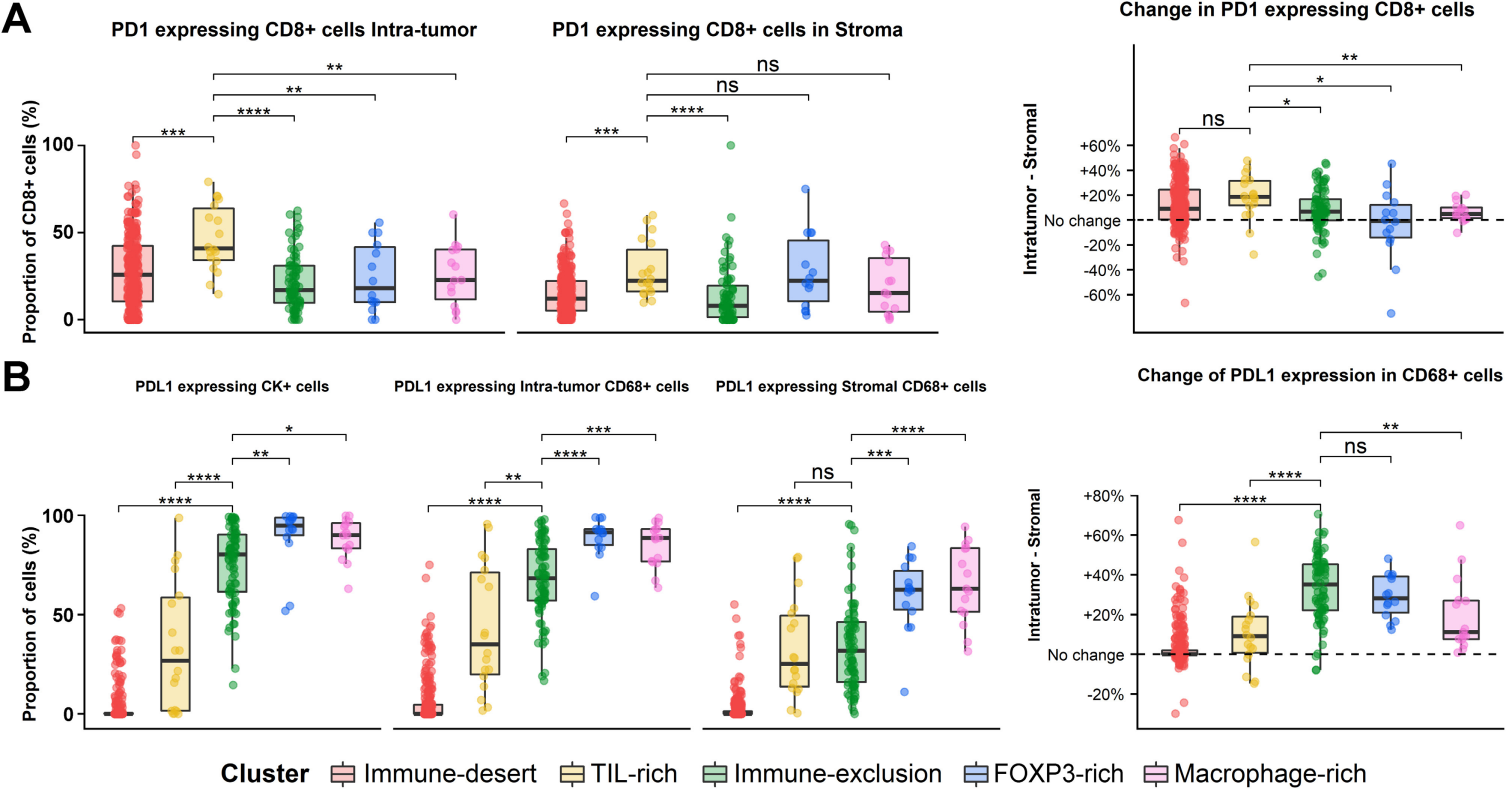
Cell phenotyping results. (A) Left panel: the proportion of PD‐1‐expressing CD8^+^ T‐cells in the tumor and stromal compartments across clusters. Right panel: PD‐1 percentage change across compartments and clusters. Positive values represent increased expression in the tumor compartment. Negative values represent increased expression in the stroma. (B) Left panel: the proportion of PD‐L1^+^ cells in tumor and CD68^+^ macrophage compartments across clusters and compartments. Right panel: change of PD‐L1 expression percentage in CD68^+^ macrophages across clusters. Positive values represent increased expression in the tumor compartment. Negative values represent increased expression in the stroma. Two‐sided *t*‐tests were used for comparisons.

The proportion of CD8^+^ T‐cells expressing PD‐1 was measured in all TMA cores. TIL‐rich tumors displayed the highest overall PD‐1 expression and significantly more CD8^+^ T‐cells expressing PD‐1 in the tumor and stromal compartment compared with the other clusters (Figure 2A). Except for the FOXP3‐rich cluster, all immune clusters demonstrated higher PD‐1 expression levels in intra‐tumor CD8 T‐lymphocytes compared with their stromal counterparts. This PD‐1 expression gradient was highest in TIL‐rich samples. Next, the PD‐L1 expression in tumor cells and TAMs was measured. Immune‐desert tumors showed the lowest PD‐L1 expression in both tumor cells and TAMs. Immune‐exclusion, FOXP3‐rich, and macrophage‐rich tumors displayed the highest PD‐L1 expression levels (Figure 2B). The expression of PD‐L1 in TAMs was heterogeneous in both intra‐tumor and stromal compartments and was significantly higher in macrophages located in the tumor compartment. However, the immune‐exclusion cluster showed the greatest tumor–stromal gradient.

### Spatial cellular interactions across the immune clusters

**Figure 3:**
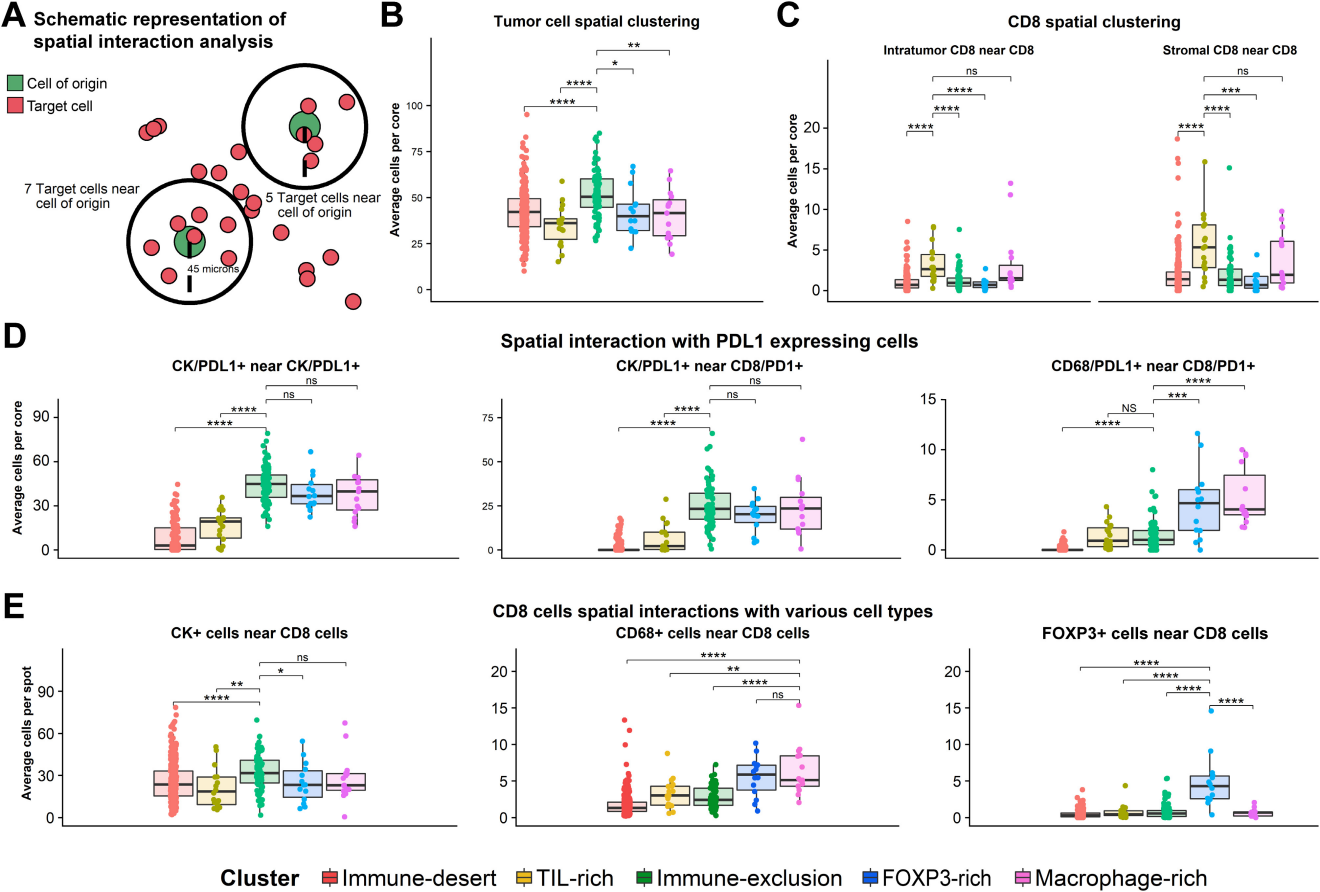
Spatial analysis results. (A) Schematic representation of the spatial interaction analysis. (B) Tumor cell spatial interaction across immune clusters. The number of tumoral cells in close contact with other malignant cells is shown. (C) CD8 T‐lymphocyte spatial clustering across immune clusters. (D) PD‐L1/PD‐1 spatial interaction results. (E) CD8 T‐lymphocyte spatial interaction with tumor, CD68^+^ macrophages, and FOXP3^+^ regulatory T‐cells. Two‐sided *t*‐tests were used for simple comparisons.

The spatial relationship among cells across the different clusters was investigated (Figure 3A). Tumor cells from the immune‐exclusion clusters displayed the highest tumor cell‐to‐tumor cell proximity independently of the PD‐L1 expression (Figure 3B,D), likely due to the highest tumor cell density found in this cluster. CD8^+^ T‐cells from TIL‐rich cores showed the highest spatial proximity (Figure 3C), indicating that these are highly inflamed cores with CD8^+^ T‐lymphocytes being more grouped in the stromal compartment. We explored the spatial interaction between CD8^+^/PD‐1^+^ T‐cells and PD‐L1‐expressing cells. Immune‐exclusion, FOXP3‐rich, and macrophage‐rich phenotypes showed the largest number of CK/PD‐L1^+^ cells close to each CD8^+^/PD‐1^+^ T‐lymphocyte. Further, CD8^+^ T‐lymphocytes belonging to immune‐exclusion samples showed limited spatial interaction with PD‐L1^+^ macrophages compared with the FOXP3‐rich and macrophage‐rich phenotypes (Figure 3D). In addition, the number of macrophages surrounding each CD8^+^ T‐cell was higher in the FOXP3‐rich and macrophage‐rich clusters (Figure 3E). As expected, CD8^+^ T‐lymphocytes from FOXP3‐rich cores showed frequent spatial proximity with FOXP3^+^ regulatory T‐cells.

### Immune phenotypes improve outcome prediction power

**Figure 4:**
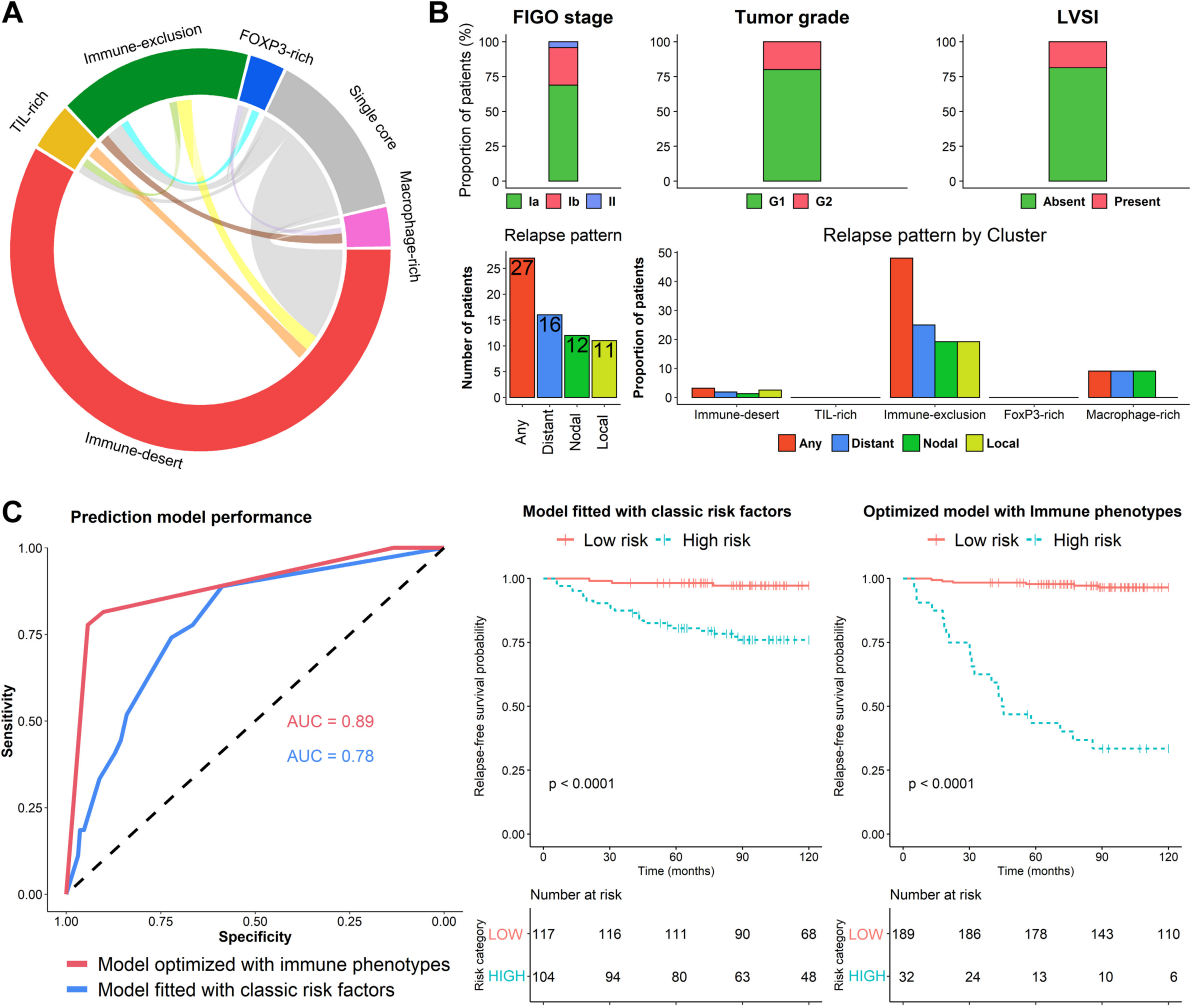
Clinical association results. (A) Circos plot displaying within‐patient immune‐phenotype variation across cores. Samples where a single core was available are linked to the grey zone denoted as ‘single core’. Non‐connected samples are considered ‘pure’. (B) Pathological variable and clinical outcomes of patients included in the analysis. LVSI, lymphovascular space invasion. (C) ROC curves and Kaplan–Meier plots for standard and immune‐optimized prediction models.

We explored the predictive power for relapse of the immune phenotype clusters. There was intra‐tumor and inter‐patient heterogeneity of immune phenotypes. Patients harboring cores belonging to the TIL‐rich, FOXP3‐rich, or macrophage‐rich clusters frequently harbored a concurrent core belonging to another phenotype (Figure 4A), possibly reflecting intra‐tumoral heterogeneity. Conversely, patients harboring the immune‐desert and immune‐exclusion phenotypes were frequently categorized as ‘pure’, where both tumor cores analyzed belonged to the same cluster type.

The mean age of the patients was 63.8 years. Most of the patients in our cohort completed at least 36 months of follow‐up. However, 6% (14/235) of patients were excluded from further analyses due to limited follow‐up. Patients included in the present study were homogeneous from a pathological standpoint. Most patients were FIGO stage IA and G1 without LVSI (Figure 4B). However, 27 of the 221 patients suffered a relapse (12%) (16 showed distant relapse, 12 lymph‐node relapse, and 11 local relapses). Fifty percent of relapses occurred before 31 months of follow‐up (p25–75 = 18.3–50.5). Patients not experiencing a relapse were followed over a median period of 120 months (p25–75 = 93.4–120). Most patients received no adjuvant radiotherapy (64.3%), while 27% of patients received either external beam radiotherapy (EBRT), vaginal brachytherapy (VBT), or both. Information regarding adjuvant radiotherapy was unavailable for 9% of the patients (supplementary material, Table S2). Thirty‐five (15.8%) and six (2.3%) patients out of the 221 patients included in the clinical outcome analysis showed MMRP deficiency and *POLE* mutations, respectively.

In sharp contrast with the other immune clusters, patients harboring the immune‐exclusion phenotype showed an increased probability of adverse outcome. Out of the 52 patients demonstrating this cluster, 25 experienced a relapse (48%). The increased risk of relapse in patients harboring the immune‐exclusion phenotype was independent of the type of relapse experienced. The proportion of patients experiencing a relapse was higher when tumors presented both cores classified as immune‐exclusion.

We optimized a predictive model using immune phenotypes. The resulting model retained immune phenotype variables alone and demonstrated an average misclassification error of 0.077 in the cross‐validation process. We tested the prediction capacity of this model against a predictive model fitted with clinicopathological variables alone. The AUC of the immune‐optimized model was considerably higher (0.89 versus 0.78). In addition, our bootstrap analysis revealed an improved prediction capacity of the alternative model over the basal model in 9,550 out of the 10,000 random sub‐samples (supplementary material, Figure S3). The p5–95 AUC range for the optimized model was 0.83–0.94, as opposed to 0.71–0.85 for the reference model. Following model fitting, high/low‐risk cohort stratification was performed using the Youden index. Cohort stratification according to the reference model showed a sensitivity of 0.89 and a specificity of 0.59 (supplementary material, Table S3). Stratification according to the optimized model resulted in a sensitivity of 0.78 and a specificity of 0.94 for relapse prediction. The higher specificity of the optimized model resulted in a higher positive predictive value (0.66 versus 0.23) while demonstrating comparable negative predictive values (0.97 for both models). Kaplan–Meier analysis demonstrated the improved stratifying ability of the optimized model for relapse‐free survival (Figure 4C). Further, as shown in supplementary material, Figure S4, subgroup analysis demonstrated that the immune‐phenotype optimized model outperformed the reference model in patients with and without MMRP deficiency (AUC 0.86 versus 0.79 and 0.91 versus 0.59, respectively), and in patients with and without adjuvant radiotherapy (AUC 0.93 versus 0.82 and 0.88 versus 0.55, respectively).

### Validation studies of the immunofluorescence results on whole‐slide tumor tissue

**Figure 5:**
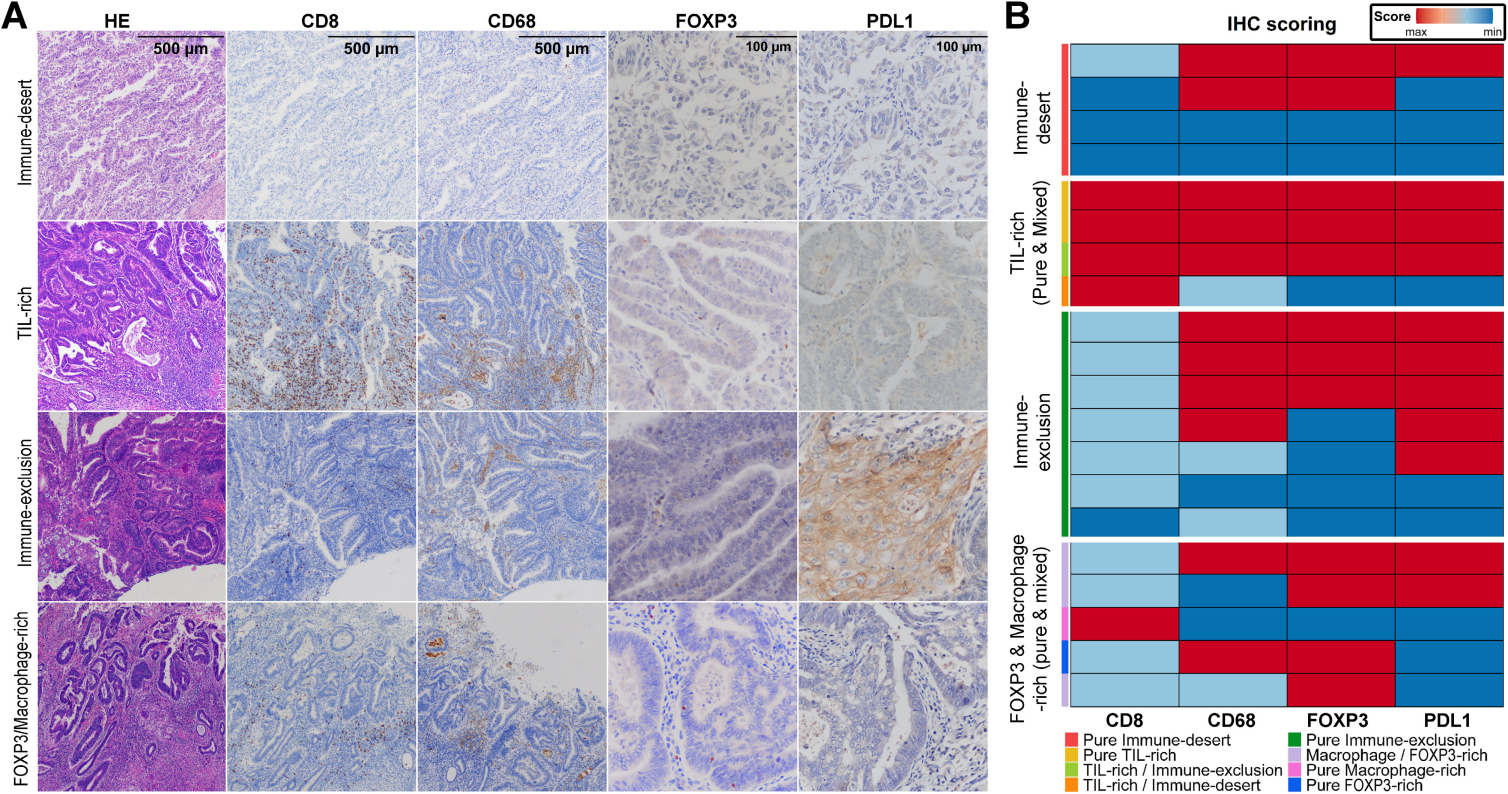
IHC validation. (A) Representative images for IHC markers across phenotypes taken at 100× magnification (HE, CD8, CD68) and 400× magnification (FOXP3 and PD‐L1). HE, hematoxylin and eosin. Scale bars are shared across columns. (B) Tile plot showing immunohistochemistry scoring results. The side color bar presents the immune phenotype identity of a case.

Finally, we explored if the immune phenotypes identified using quantitative multiplex immunofluorescence could be validated by whole‐slide single‐marker IHC scoring (Figure 5A). First, we used intra‐tumoral immune‐cell density metrics to fit a tree model that could stratify samples into different clusters with high accuracy (supplementary material, Figure S5). Intra‐tumoral PD‐L1, CD8, CD68, and FOXP3 demonstrated high classification power, as the model fitted with these variables was able to accurately identify all immune phenotypes (76–100% precision). Further, a simplified tree model (fitted with intra‐tumoral CD8 and PD‐L1 expression) was able to correctly identify immune‐desert, TIL‐rich, and immune‐exclusion cases, albeit with lower precision (56–96%) (supplementary material, Figure S5).

Our IHC results validated the immunofluorescence results (Figure 5B). High intra‐tumoural CD8^+^ T‐cell infiltration was observed in all tumors harboring the TIL‐rich immune phenotype. The immune‐desert and immune‐exclusion tumors showed low CD8^+^ T‐cell infiltration. Although variability was observed, at least minimal CD8 infiltration in the hottest spot was identified in most tumors. Except for isolated samples, most tumors showed a certain degree of macrophage infiltration. Unexpectedly, CD68^+^ macrophage infiltration scored by IHC showed a similar distribution across clusters. In addition, although most tumors showed at least focal FOXP3^+^ regulatory T‐cell infiltration, all cases harboring the FOXP3‐rich phenotype scored positive in the present analysis. Finally, although focal, PD‐L1 expression could be demonstrated by IHC in most cases of the immune‐exclusion phenotype.

Finally, we compared the prediction performance of these individual markers measured by quantitative multiplex immunofluorescence against the prediction model. Except for PD‐L1‐expressing tumor cells (which showed comparable results), all other single immune markers showed a worse AUC for relapse prediction (supplementary material, Figure S6).

## Discussion

Evidence supports a role for the immune system in influencing the initiation and maintenance of endometrial carcinogenesis [26, 27, 28]. Past studies have focused on the association between endometrial cancer outcomes and single immune‐cell populations [29, 30, 31, 32]. More comprehensive approaches have used gene signatures to predict clinical outcome [33, 34, 35, 36, 37]. However, little is known about the interactions between tumor cells and the microenvironment in low‐grade, early‐stage endometrial carcinomas. Past efforts to characterize the TIME of endometrial tumors have included a very heterogeneous group of patients with diverse grade, FIGO stage, and molecular subtypes. Therefore, it is difficult to translate these results into clinical practice.

In contrast to earlier studies, we analyzed the immune phenotype of a clinically homogeneous group of patients diagnosed with low‐grade, early‐stage endometrioid tumors. By selecting a specific subgroup of patients, the results are more meaningful from a clinical standpoint. Five different patterns of immune infiltration have been identified, including the classical immune‐depleted and infiltrated tumors [10]. Although the identification of the immune infiltration patterns was performed agnostic to the patient outcome, they have shown a good correlation with the likelihood of relapse. Clusters with a relatively high density of TILs, FOXP3^+^ regulatory T‐cells, and TAMs, respectively, were all associated with a better prognosis. These results are in line with previous studies reporting a positive association between CD8^+^ T‐lymphocytes and outcome [30, 38]. The fact that previous studies suggested a positive association between regulatory T‐cells and worse outcome [39] should be interpreted taking into account differences in the clinical characteristics of the cohorts studied and the methods used to quantify regulatory T‐cells. Further, one study analyzing chemotherapy‐treated stage III colorectal carcinomas found improved outcomes for regulatory T‐cell tumors [40].

The immune phenotype associated with worse prognosis was characterized by low TIL infiltration and a relatively high density of PD‐L1‐expressing tumor cells. Studies into the association between PD‐L1 expression and clinical outcomes have shown conflicting results in endometrial cancer. Even if high‐grade and advanced tumors show higher PD‐L1 expression [41, 42, 43], this has also been linked to improved outcomes [44]. Our results suggest that the prognostic impact of PD‐L1 expression is influenced by tumor type and stage. Given that it is associated with worse prognosis, patients harboring this phenotype could potentially benefit from adjuvant immunotherapy treatment.

The immune phenotypes found in the present study showed a weak correlation with molecular subtypes. We found an enrichment of MMRP deficiency in samples from TIL‐rich and immune‐exclusion phenotypes. The fact that an immune‐exclusion phenotype is enriched in MMRP‐deficient tumors is supported by previous studies analyzing the singular immune microenvironment of MMRP‐deficient endometrial cancer compared with MMRP‐deficient tumors from other locations [45]. In addition, the correlation between the immune phenotypes and *POLE* or *CTNNB1* mutation status was non‐significant.

The immune‐cell phenotyping performed in the present study has enabled us to explore the PD‐1/PD‐L1 axis across the tumor and stromal compartments. Tumors showing high TIL infiltration displayed increased PD‐1 expression in the tumor compartment, a finding that has been reported in melanoma and renal cell carcinomas [46, 47]. This could reflect lymphocyte transition to an exhausted phenotype after recognition of tumor. On the other hand, TAMs infiltrating tumors from the immune‐exclusion phenotype showed increased PD‐L1 expression compared with their stromal counterparts. This intense TAM polarization pattern was not found in other immune clusters. This finding reflects the increased immunosuppressive macrophage polarizing abilities of a subset of these tumors.

We have explored the heterogeneity of cell–cell spatial interactions. First, we quantified the increased probability of a CD8^+^/PD‐1^+^ T‐lymphocyte to spatially engage its PD‐1‐ligand on tumor cells in patients displaying immune exclusion. In contrast, CD8^+^/PD‐1^+^ T‐cells from other immune phenotypes show a higher probability of engaging PD‐L1 in macrophages. Second, our study has revealed the increased tumor cell burden of immune‐exclusion cases, and the consequently high numbers of tumor cells in close contact with TILs. Third, we have demonstrated that immune profiles characterized by a high number of macrophages or FOXP3^+^ regulatory T‐cells also display increased spatial proximity between these cell populations and CD8^+^ T‐lymphocytes. This approach has already been able to show potential clinical implications in renal cell carcinoma [47]. To date, a similar analysis has not been performed on endometrial tumors. Our results serve as a proof of concept that cell–cell spatial interaction adds a layer of microenvironment complexity.

Lastly, our data suggest that the TIME could be used to improve prediction of clinical outcomes. This has already been explored and validated with great success in colorectal cancer [12]. For endometrial cancer patients, management decisions are based on FIGO staging, tumor grade, and presence of LVSI. Although molecular subtypes have been found to add prognostic information to classic risk factors [18], they show great overlap with the histologic types and tumor grades already included in the classification schemes. Further, *POLE* mutation that is associated with improved prognosis in high‐grade tumors is less frequently found in low‐grade, early‐stage endometrial cancers as suggested here and by previous studies [4, 48, 49]. Thus, its use as a biomarker in early‐stage endometrial cancer is limited. The prognostic model presented in the present study was fitted independent of the molecular subtypes, which could be an advantage in limited resource settings. In addition, the fact that low‐grade, early‐stage endometrial cancer patients show low relapse rates indicates that highly specific prognostic biomarkers are required to avoid overtreatment. Noteworthy, our prediction model showed high specificity and positive predictive value. Although further validation in independent cohorts is required, our data suggest that a simple immune‐microenvironment characterization (based on broad immune‐cell populations and phenotypes) outperforms classic risk factors in predicting relapse in a highly specific subset of patients. Importantly, this improved risk stratification was independent of adjuvant radiotherapy treatment or MMRP status, both of which are potential confounders in our analysis.

Even though our IHC validation suggests that immunofluorescence data can be partially recapitulated using a single‐marker assay, some limitations are worth mentioning. First, the limited sample size used in our validation warrants further studies to improve the generalizability of the results. Second, only intra‐tumoral markers were considered in the IHC evaluation protocol. Third, there are inherent differences between naked eye IHC scoring and quantitative immunofluorescence that limit reproducibility. Finally, while immunofluorescence was carried out on TMAs, IHC validation was performed on serial whole‐slide tissue sections, which may partially explain the discrepancies in the results. Further, the scoring in the TIME in diverse tumor regions (infiltration border or tumor core), as used in other scenarios [12], could further improve the predictive power. Future validation of the present results could overcome these limitations by increasing the sample size, using digital pathology to quantify populations in a non‐biased manner in tumor and stromal compartments, and by performing multiplex IHC instead of serial IHC sections to properly assess spatially co‐localization of immune populations.

In summary, we have demonstrated heterogeneity in the TIME of low‐grade, early‐stage endometrial carcinoma. We have identified five different patterns of immune infiltration. These phenotypes are associated with specific cell phenotypes and cell–cell spatial interactions. In addition, we have been able to harness this diversity to improve clinical outcome predictions.

## Author contributions statement

ALJ, MVE, DH and CEA conceived the study. MVE, MEB, DJS, IRC, DGR, EK, VG and AP carried out experiments. ALJ, MVE and IvM performed data analysis. ALJ, MVE, IgM, DH and CEA performed data interpretation. ALJ, MVE, IgM, DH and CEA wrote the manuscript.

## Supporting information


Supplementary materials and methods
**Figure S1.** Multiplex staining protocols and validation workflow
**Figure S2.** (A) Correlation matrix showing Pearson correlations between the different immune parameters measured. (B) Percentage of cores across immune phenotypes belonging to mismatch repair protein (MMRP)‐deficient tumors, *POLE*‐mutated tumors, or *CTNNB1*‐mutated tumors
**Figure S3.** (A) Results of basal and immune‐optimized logistic regression models for relapse outcome. (B) Bootstrapped analysis of model AUC performance for basal (fitted with classic pathologic variables) and immune‐optimized models
**Figure S4.** Model prediction performance stratified by mismatch repair protein status and adjuvant radiotherapy treatment
**Figure S5.** Decision tree resulting from applying the recursive partitioning algorithm on intra‐tumor measured immune variables
**Figure S6.** ROC curves prediction ability of relapse‐free survival for individual immune markers (maximum value per patient) and model optimized with immune phenotypes
**Table S1.** List of immune related variables included in clustering analysis
**Table S2.** Clinicopathological information of patients included in and excluded from clinical outcome analysis
**Table S3.** Comparison of model risk‐classification stratified by clinical outcome

**Guideline S1.** IHC immune biomarker reporting guidelines (referred to in Supplementary materials and methods)

## Data Availability

Data and code used in the present study can be found at https://github.com/Alvaro-LJ/Immune_env_Endometrial_Cancer.
