# Supplementary material for: The association between the tumor immune microenvironments and clinical outcome in low‐grade, early‐stage endometrial cancer patients
Source: J Pathol. 2022 Oct 25;258(4):426–36. doi: 10.1002/path.6012 (PMC9828119; doi:10.1002/path.6012)
Supplement: Supplementary file 1 — Supplementary materials and methods Figure S1. Multiplex staining protocols and validation workflow Figure S2. (A) Correlation matrix showing Pearson correlations between the different immune parameters measured. (B) Percentage of cores across immune phenotypes belonging to mismatch repair protein (MMRP)‐deficient tumors, POLE‐mutated tumors, or CTNNB1‐mutated tumors Figure S3. (A) Results of basal and immune‐optimized logistic regression models for relapse outcome. (B) Bootstrapped analysis of model AUC performance for basal (fitted with classic pathologic variables) and immune‐optimized models Figure S4. Model prediction performance stratified by mismatch repair protein status and adjuvant radiotherapy treatment Figure S5. Decision tree resulting from applying the recursive partitioning algorithm on intra‐tumor measured immune variables Figure S6. ROC curves prediction ability of relapse‐free survival for individual immune markers (maximum value per patient) and model optimized with immune phenotypes Table S1. List of immune related variables included in clustering analysis Table S2. Clinicopathological information of patients included in and excluded from clinical outcome analysis Table S3. Comparison of model risk‐classification stratified by clinical outcome [file PATH-258-426-s001.docx]

**The association between the tumor immune microenvironments and clinical outcome in low-grade, early-stage endometrial cancer patients**

Á López-Janeiro *et al. J Pathol* <https://doi.org/10.1002/path.6012>

**Supplementary materials and methods**

**Supplementary Figures S1–S6**

**Supplementary Tables S1–S3**

Reference numbers refer to the main text list

**Supplementary materials and methods**

*Multiplex immunofluorescence*

Four-micrometer-thick sections from TMAs were deparaffinized, and antigen retrieval was performed using heat-induced antigen retrieval with high pH (pH 9) solutions. Samples were stained using primary antibodies targeting cytokeratin (pan-CK) (1:150, AE1/AE3; Novus Biologicals, Littleton, CO, USA), CD8 (1:150, 4B11; Bio-Rad, Hercules, CA, USA), CD68 (1:75, PG-M1; Dako-Agilent, Santa Clara, CA, USA), FOXP3 (1:50, 236A/E7; Abcam, Cambridge, UK), PD-1(1:300, ERP4877, Abcam), and PD-L1 (1:300, E1L3N; Cell Signaling Technology, Danvers, MA, USA) followed by tyramide signal amplification (TSA) visualization with fluorophores Opal-690, Opal-540, Opal-620, Opal-570, Opal-650, and Opal-520 (Akoya Biosciences, Marlborough, MA, USA), respectively. All steps were performed using the LabSat^®^ Research platform (Lunaphore Technologies, Tolochenaz, Switzerland), an automated tissue-staining instrument for rapid immunostaining which utilizes a microfluidic technology for the rapid and uniform delivery of reagents to tissue samples [16,17]. Each TMA section underwent sequential rounds of antibody staining, as previously described [16]. In the last round, nuclei were counterstained with spectral DAPI (Akoya Biosciences) and mounted with Faramount Aqueous Mounting Medium (Dako-Agilent).

TMA tissue sections were scanned using a PhenoImager HT (Akoya Biosciences). Tissue imaging, spectral unmixing, and image analysis were performed using inForm software (version 2.4.8, Akoya Biosciences), as described previously [15,16]. Autofluorescence was determined on an unstained tumor section. Tissue segmentation algorithms based on cytokeratin were used to identify tumor islands, stroma areas, and regions of non-interest. Cell segmentation algorithms based on nuclear detection (DAPI) were used to identify each nucleus. Each detected nucleus was then expanded to give an approximation of the full cell area, constrained by the distance and the proximity of neighboring cells. Algorithm classifiers were trained separately for each cell marker using the features generated earlier by having an experienced pathologist annotate regions in a subset of images from a training set, with interactive feedback on classification performance provided during training in the form of a markup image, as described previously [15,16]. Based on the fluorescence panel, cells were further subclassified as CD68^+^, CD8^+^, FOXP3^+^, and CK^+^. Subpopulations within CD68^+^, CD8^+^ T, and CK^+^ cells were then classified by the presence and absence of the additional markers: PD-1 and PD-L1. CK was used to visualize tumor cells. The nearest neighbor distances between various cell types were studied to characterize the spatial organization of the cell populations within an area of a 45 μm radius. The spatial densities for different cells type were estimated using a *k* nearest neighbors (*k*NN) model. Quality control steps were implemented. TMA cores showing frank detachment from the slide (less than 50% of the expected core area) and those with low tumor content (tumor occupying less than 20% of the total area) were discarded.

*Clustering analysis*

To identify immune profiles, we performed hierarchical clustering using the immune population densities (supplementary material, Table S1). Hierarchical clustering was performed using the Manhattan distance and the Ward.D2 agglomerative algorithm from base R. A previously published pipeline was used to determine the optimal number of clusters to perform dendrogram splitting [50]. In brief, 100 bootstrapped samples were generated from our original data set. Hierarchical clustering was applied to build a dendrogram in each of the subsamples. We sequentially split the resulting dendrograms in *K* = 2, to *K* = 20 clusters. For each of the clustering results, we calculated Davies–Boulding and Goodall scores as cluster robustness metrics. Results were manually revised and the number of *K* that maximized average G1 scores while minimizing the average Davies–Boulding score was chosen. Further, cluster stability was checked by calculating the Jaccard index using the fpc R package [51].

*Immunohistochemistry*

Immunohistochemistry was performed on TMA sections using the following primary antibodies: MLH1 (prediluted, ES05, Dako-Agilent), PMS2 (prediluted, EP51, Dako-Agilent), MSH2 (prediluted, FE11, Dako-Agilent), MSH6 (prediluted, EP49, Dako-Agilent), and p53 (prediluted, DO-7, Dako-Agilent). For mismatch repair proteins, partial or complete absence of nuclear expression of any of the markers was considered abnormal. For p53 pattern determination, either diffuse intense nuclear expression, absent nuclear expression, or cytoplasmic staining was considered abnormal according to previously published guidelines [6]. Whole-slide immunohistochemistry was performed in equivocal cases.

For IHC whole-slide validation, consecutive slices from tissue blocks were stained with CD8 (prediluted, cC8/144B, Dako-Agilent), CD68 (prediluted, KP1, Dako-Agilent), FOXP3 (1:100, 86D; Biocare, Pacheco, CA, USA), and PD-L1 (1:200, E1L3, Cell Signaling Technology), as well as with hematoxylin/eosin. Slides were reviewed by a pathologist who was blinded to the identity of the tumor immune phenotype. The pathologist was requested to score each of the markers in a semi-quantitative manner using a two or three-tiered scale (supplementary material, Guideline S1). The pathologist performed two scoring rounds. Discrepant cases were resolved in a third decision round.

To simplify the stratification tool, we also fitted a recursive partitioning algorithm using tumoral CK/PD-L1 and intra-tumoral CD8^+^ cell densities.

Finally, we also compared the relapse prediction ability of the prediction model against the individual immune variables. To this end, we first annotated the maximum QIF expression of selected markers per patient. After, we calculated the AUC for relapse prediction for each of these markers and compared it with the AUC of the optimized model.

*Data analysis*

Data analyses were carried out using R (4.0.1) and R studio [25]. Circos plots and correlation matrices were graphed using *circlize* and *corrplot* packages [52,53]. Heatmaps were graphed using the *ComplexHeatmap* package [54]. All other graphs were built using *ggplot2*, *cowplot*, and *magick* packages [55–57].

**Supplementary Figures S1–S6**


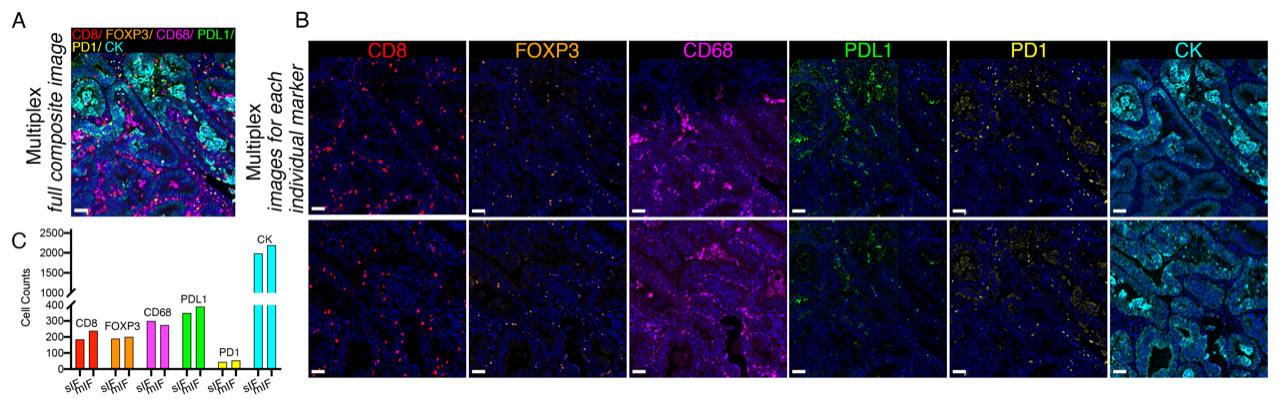


**Figure S1.** Multiplex staining protocols and validation workflow. (A) Spectrally unmixed composite image of FFPE tissue section from low-grade endometrial cancer stained against CD8 (red), FOXP3 (orange), CD68 (magenta), PD-L1 (green), PD-1 (yellow), CK (cyan), and DAPI (blue). (B) Images for each of the individual markers of the multiplex panel are shown. (B, C) Multiplex immunofluorescence (mIF) images were compared against the corresponding fluorescence channel from singleplex IF (sIF) images from sequential tissue sections (bar charts). The total number of cells for each marker comparing singleplex IF against the corresponding fluorescence channel from the multiplex IF on sequential FFPE sections was used for optimizing the seven-color multiplex immunolabelling protocol. Scale bars: 50 μm.


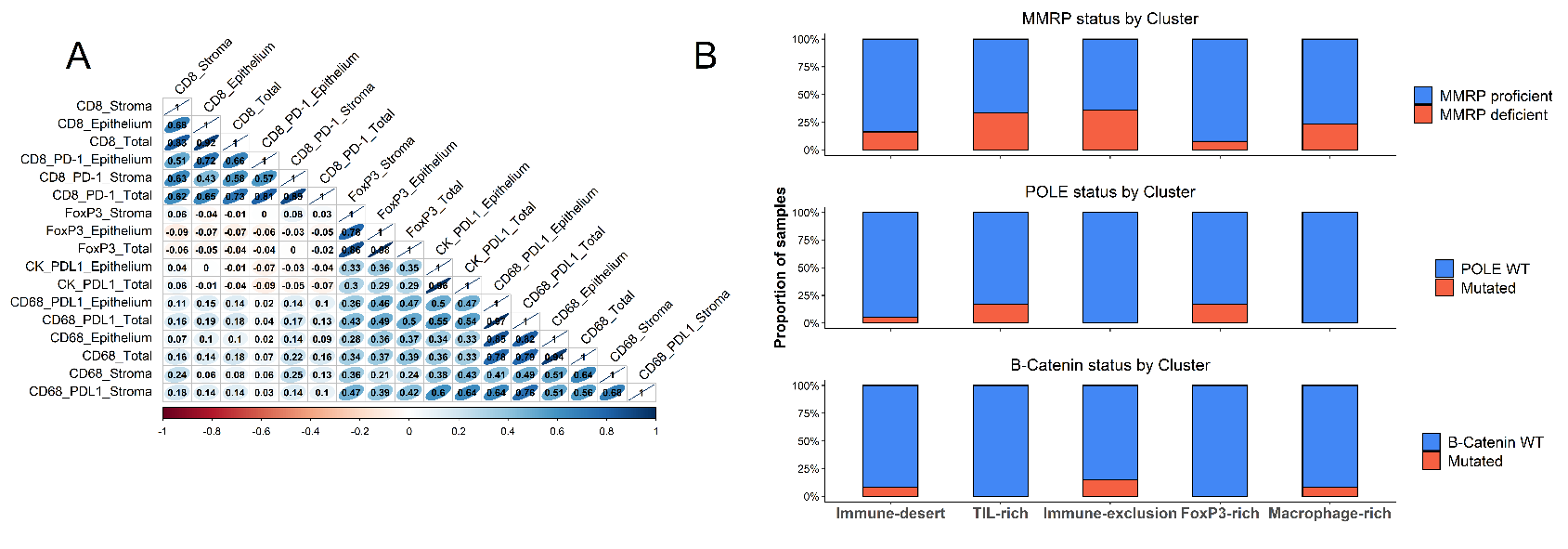


**Figure S2.** (A) Correlation matrix showing Pearson correlations between the different immune parameters measured. (B) Percentage of cores across immune phenotypes belonging to mismatch repair protein (MMRP)-deficient tumors, *POLE*-mutated tumors, or *CTNNB1*-mutated tumors.


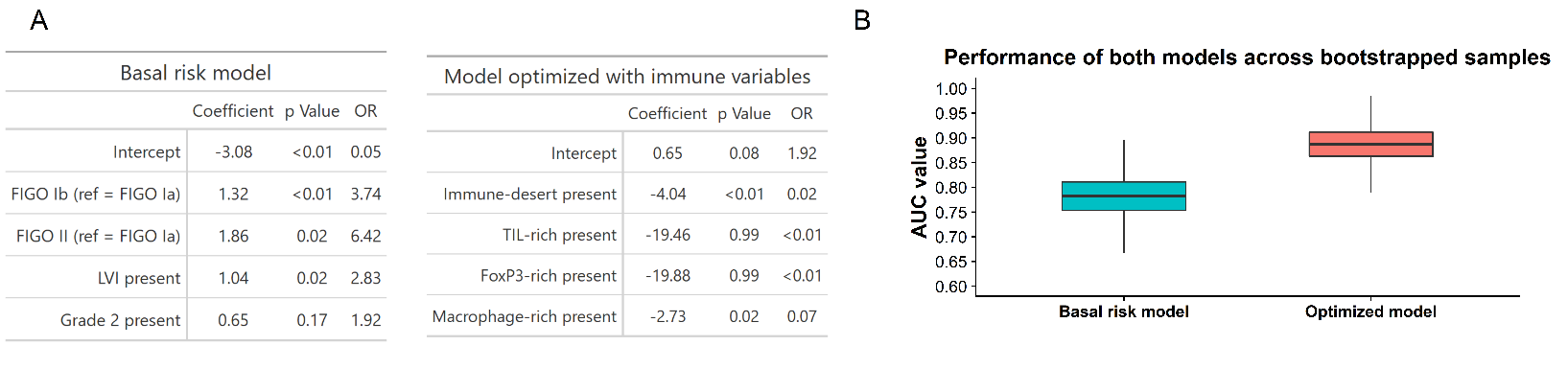


**Figure S3.** (A) Results of basal and immune-optimized logistic regression models for relapse outcome. (B) Bootstrapped analysis of model AUC performance for basal (fitted with classic pathologic variables) and immune-optimized models.


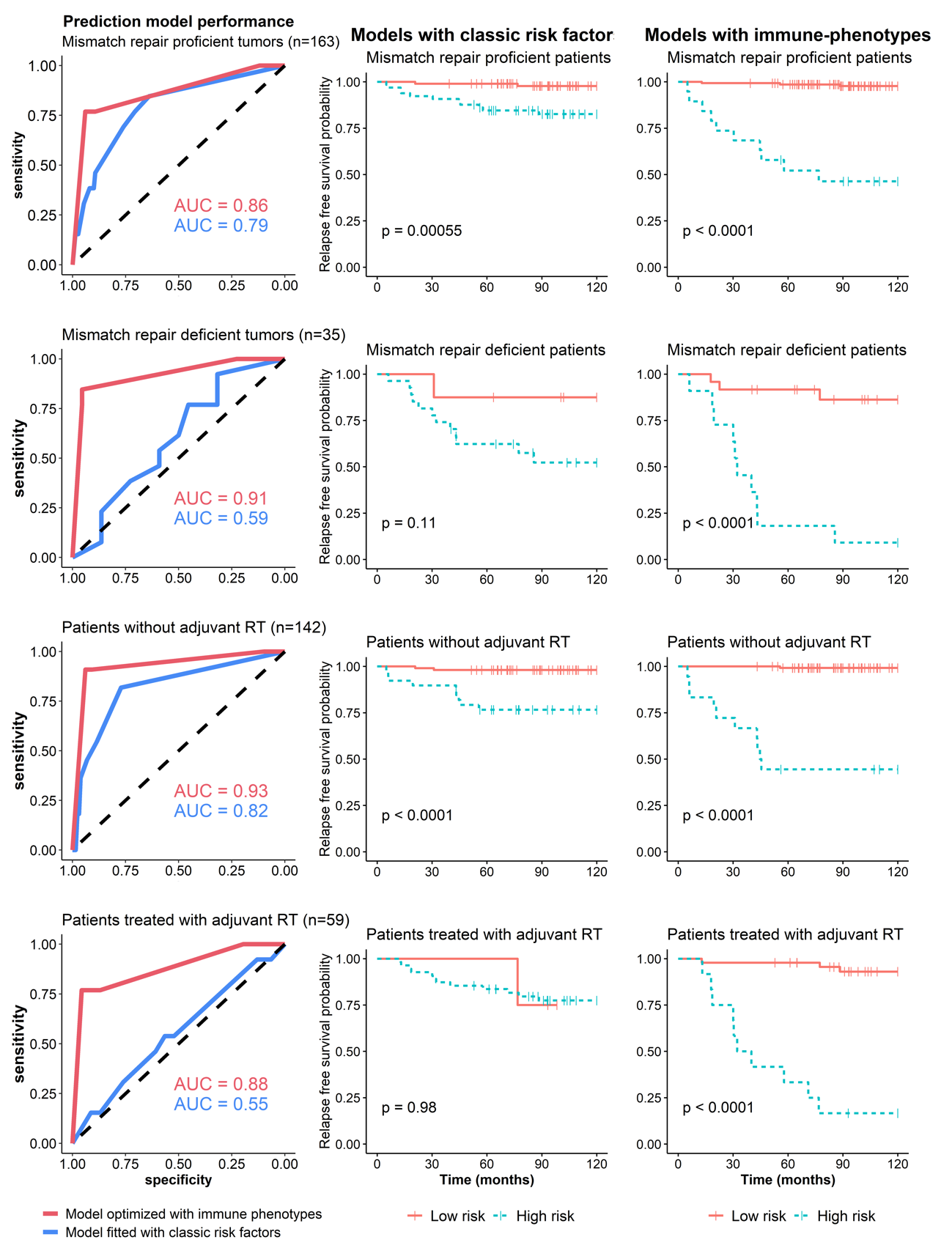


**Figure S4.** Model prediction performance stratified by mismatch repair protein status and adjuvant radiotherapy treatment. Left-side panels: ROC curves denoting relapse-free survival prediction ability for basal (fitted with classic pathologic variables) and immune-optimized models stratified by mismatch repair protein status and adjuvant treatment. Center panels: KM plots for patients classified as low or high risk according to basal model results and stratified by mismatch repair protein status and adjuvant treatment. Right-side panels: KM plots for patients classified as low or high risk according to optimized model results and stratified by mismatch repair protein status and adjuvant treatment.

**
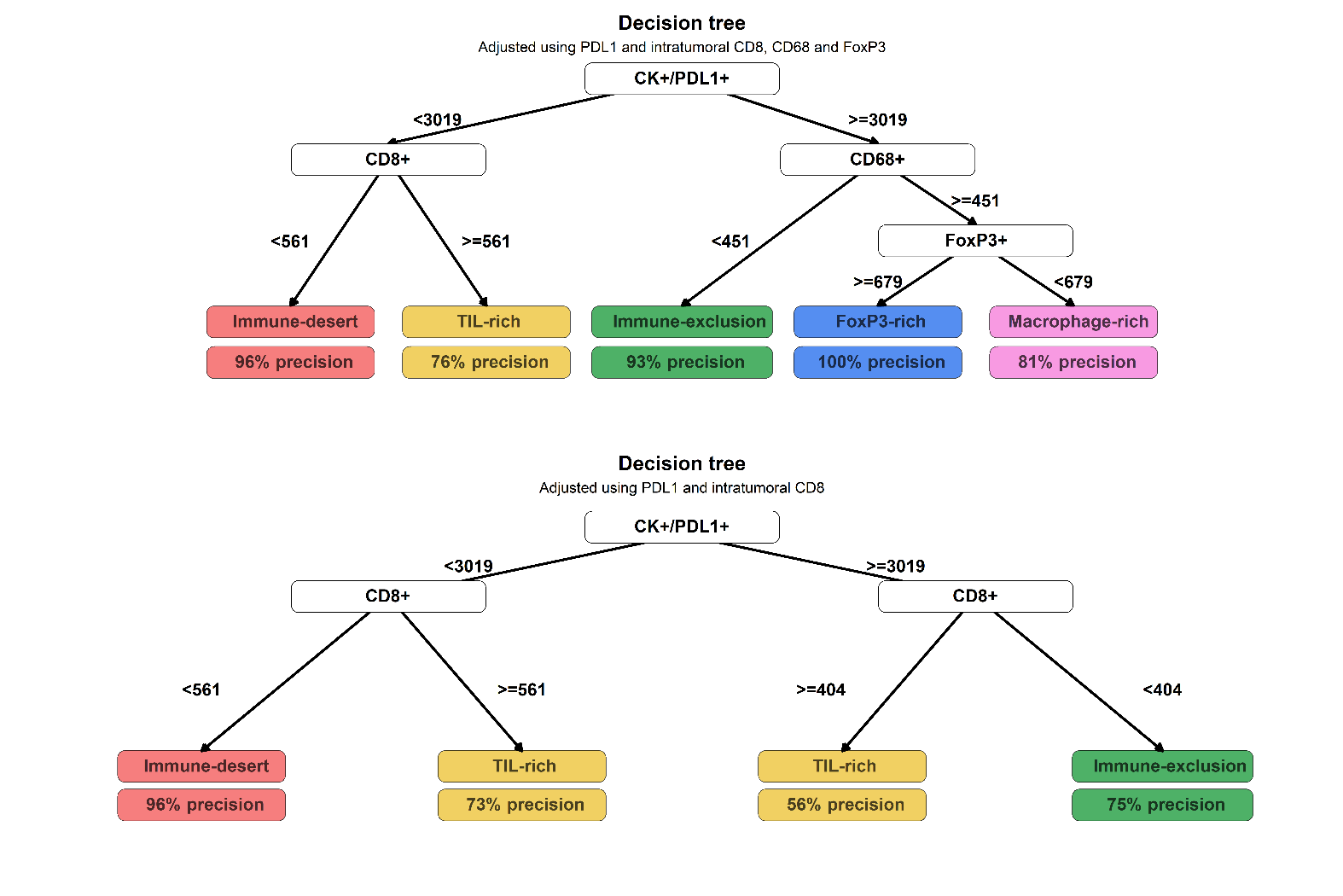
Figure S5.** Decision tree resulting from applying the recursive partitioning algorithm on intra-tumor measured immune variables. Splitting cut-off numbers represent cell densities measured as cells/mm^2^. (A) Decision tree adjusted using PD-L1, CD8, CD68, and FoxP3. (B) Decision tree adjusted using only PD-L1 and CD8.

**
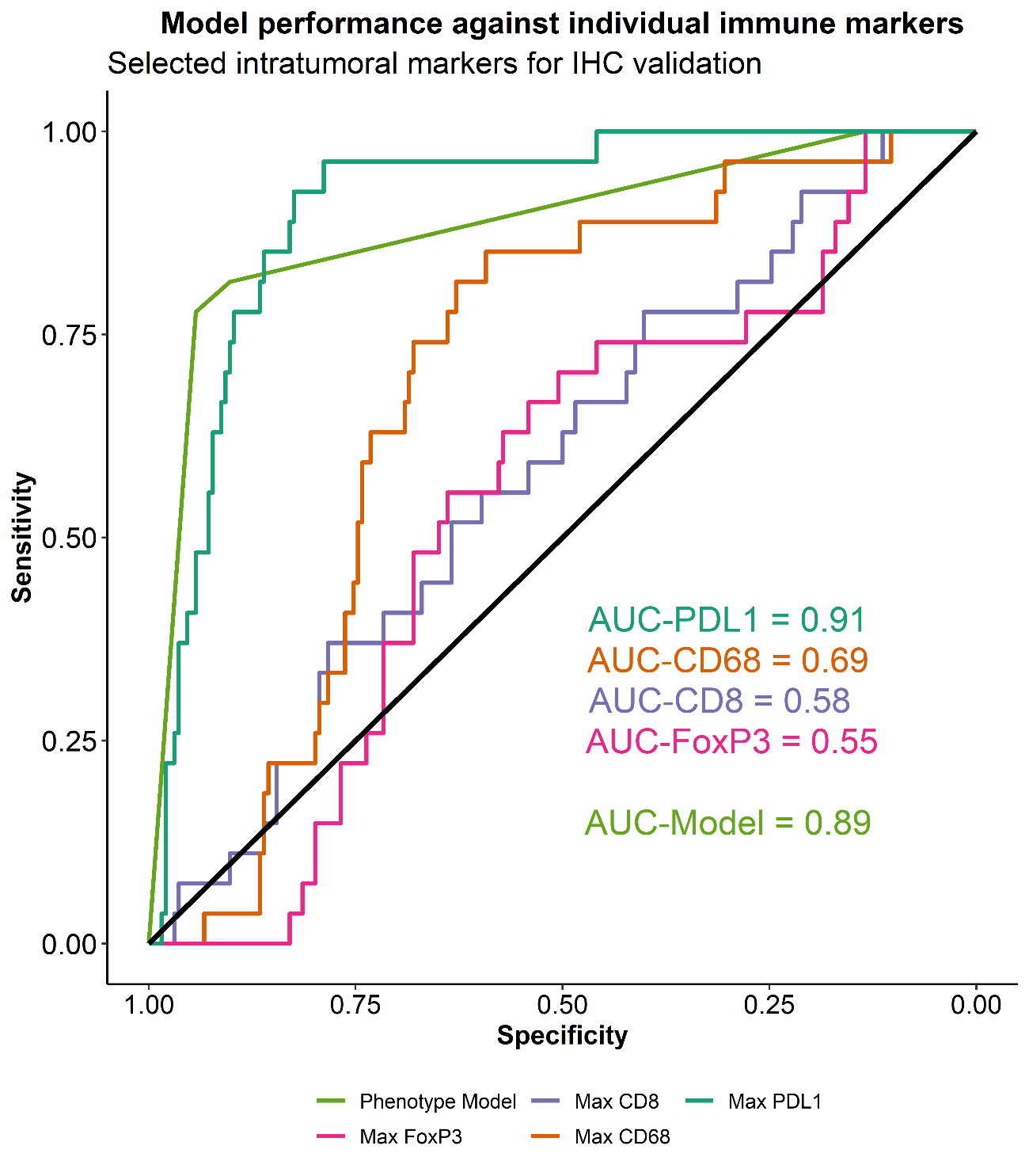
Figure S6.** ROC curves prediction ability of relapse-free survival for individual immune markers (maximum value per patient) and model optimized with immune phenotypes.

|  | **Variable name** | **Description** |
| --- | --- | --- |
| Cytotoxic lymphocytes | CD8_Total | CD8^+^ cells per mm^2^ in the whole spot |
|  | CD8_Epithelium | CD8^+^ cells per mm^2^ in tumor compartment |
|  | CD8_Stroma | CD8^+^ cells per mm^2^ in stromal compartment |
| Macrophages | CD68_Total | CD68^+^ cells per mm^2^ in the whole spot |
|  | CD68_Epithelium | CD68^+^ cells per mm^2^ in tumor compartment |
|  | CD68_Stroma | CD68^+^ cells per mm^2^ in stromal compartment |
| Tregs | FoxP3_Total | FoxP3^+^ cells per mm^2^ in the whole spot |
|  | FoxP3_Epithelium | FoxP3^+^ cells per mm^2^ in tumor compartment |
|  | FoxP3_Stroma | FoxP3^+^ cells per mm^2^ in stromal compartment |
| PD1^+^ T cells | CD8_PD-1_Total | PD1^+^/CD8^+^ cells per mm^2^ in the whole spot |
|  | CD8_PD-1_Epithelium | PD1^+^/CD8^+^ cells per mm^2^ in tumor compartment |
|  | CD8_PD-1_Stroma | PD1^+^/CD8^+^ cells per mm^2^ in stromal compartment |
| PDL1 expression | CD68_PDL1_Total | PDL1^+^/CD68^+^ cells per mm^2^ in the whole spot |
|  | CD68_PDL1_Epithelium | PDL1^+^/CD68^+^ cells per mm^2^ in tumor compartment |
|  | CD68_PDL1_Stroma | PDL1^+^/CD68^+^ cells per mm^2^ in stromal compartment |
|  | CK_PDL1_Epithelium | PDL1^+^/CK^+^ cells per mm^2^ in tumor compartment |
|  | CK_PDL1_Total | Overall PDL1^+^/CK^+^ cells independent of tumor mask |

**Supplementary Tables S1–S3**

**Table S1.** List of immune related variables included in clustering analysis.

**Table S2.** Clinicopathological information of patients included in and excluded from clinical outcome analysis.

| **Included in clinical outcome analysis (*n* = 221)** | |
| --- | --- |
| Age (years, mean, p25–75) | 63.8 (56–72) |
| FIGO stage | IA = 152 (68.8%)  IB = 60 (27.1%)  II = 9 (4.1%) |
| FIGO grade | G1 = 177 (80%)  G2 = 44 (20%) |
| Presence of lymphovascular invasion | Absent = 180 (81.4%)  Present = 41 (18.6%) |
| Mismatch repair protein status | Proficient = 163 (73.76%)  Deficient = 35 (15.8%)  Not available = 23 (10.4%) |
| POLE (exons 9-11-13-14) mutation status | Wild type = 123 (55.7%)  Mutated = 6 (2.7%)  Not available = 92 (41.6%) |
| Molecular subgroup | Copy number low/endometrioid = 89 (40.2%)  MMRPd = 22 (10%)  *POLE* mutated = 5 (2.3%)  Double classifiers (POLE/MMRPd) = 1 (0.4%)  Not available = 104 (47.1%) |
| Adjuvant radiotherapy | No radiotherapy = 142 (64.3%)  EBRT = 4 (1.8%)  VBT = 23 (10.4%)  EBRT + VBT = 32 (14.5%)  Not available = 20 (9%) |
| **Excluded from clinical outcome analysis (*n* = 14)** | |
| Age (years, mean, p25–75) | 69.7 (69.25–77.5) |
| FIGO stage | IA = 9 (64.3%)  IB = 2 (14.3%)  II = 3 (21.4%) |
| FIGO grade | G1 = 8 (57.1%)  G2 = 6 (42.9%) |
| Presence of lymphovascular invasion | Absent = 12 (85.7%)  Present = 2 (14.3%) |
| Mismatch repair protein status | Proficient = 7 (50%)  Deficient = 5 (35.7%)  Not available = 2 (14.3%) |
| POLE (exons 9-11-13-14) mutation status | Wild type = 11 (78.6%)  Mutated = 1 (7.1%)  Not available = 2 (14.3%) |
| Molecular subgroup | Copy number low/endometrioid = 4 (28.6%)  MMRPd = 5 (35.7%)  *POLE* mutated = 1 (7.1%)  Double classifiers (POLE/MMRPd) = 0 (0%)  Not available = 4 (28.6%) |
| Adjuvant radiotherapy | No radiotherapy = 6 (42.9%)  EBRT = 0 (0%)  VBT = 0 (0%)  EBRT + VBT = 1 (7.14%)  Not available = 7 (50%) |
| MMRPd = mismatch repair proteins deficient; EBRT = external beam radiation therapy; VBT = vault brachytherapy. | |

**Table S3.** Comparison of model risk-classification stratified by clinical outcome.

| **NO RELAPSE (*n* = 194)** |  | **OPTIMIZED MODEL** | | | |  |
| --- | --- | --- | --- | --- | --- | --- |
|  |  | LOW RISK | | HIGH RISK | |  |
| **BASAL MODEL** | LOW RISK | 109 (56.2%) | | 5 (2.6%) | |  |
|  | HIGH RISK | 74 (38.1%) | | 6 (3.1%) | |  |
| **RELAPSE (*n* = 27)** |  | **OPTIMIZED MODEL** | | | |  |
|  |  | LOW RISK | | HIGH RISK | |  |
| **BASAL MODEL** | LOW RISK | 0 (0%) | | 3 (11.1%) | |  |
|  | HIGH RISK | 6 (22.2%) | | 18 (66.7%) | |  |
|  | METRIC | | | | | |
| **MODEL** | **Sensitivity** | **Specificity** | **PPV** | | **NPV** | |
| BASAL MODEL | 88.9% | 58.8% | 23.1% | | 97.4% | |
| OPTIMIZED MODEL | 77.8% | 95.3% | 65.6% | | 96.8% | |
| Basal model: model fit using classic risk factors.  Optimized model: model optimized with immune phenotypes.  PPV: positive predictive value; NPV: negative predictive value. | | | | | | |
