# Supplementary material for: The association between the tumor immune microenvironments and clinical outcome in low‐grade, early‐stage endometrial cancer patients
Source: J Pathol. 2022 Oct 25;258(4):426–36. doi: 10.1002/path.6012 (PMC9828119; doi:10.1002/path.6012)
Supplement: Supplementary file 2 — Guideline S1. IHC immune biomarker reporting guidelines (referred to in Supplementary materials and methods) [file PATH-258-426-s002.pdf]

The association between the tumor immune microenvironments and clinical outcome in low-grade, early-stage endometrial cancer patients
Á López-Janeiro *et al.* *J Pathol* DOI: https://doi.org/10.1002/path.6012

Guideline S1. IHC immune biomarker reporting guidelines.

IHC IMMUNE BIOMARKER REPORTING GUIDELINES

General considerations

- The intra-tumour compartment should be analyzed, avoiding stromal compartment scoring.
- Scoring is based on immune infiltration **densities**. NOT percentage of cells.
- Scoring should be done at the **HOT-SPOT** for each marker (100x field).
- Semi-quantitative scoring should be performed according to reference images provided below.
- Images provided are "Pseudo-IHC" reconstructions from immunofluorescence images. In addition, clones used for the present assay and for immunofluorescence differ. Therefore, please bear in mind that the staining quality of the representative images differs from the actual IHC.

CD8

CD8 GRADE 0 (0+)

Absent or very infrequent INTRA-TUMOR lymphocytes.

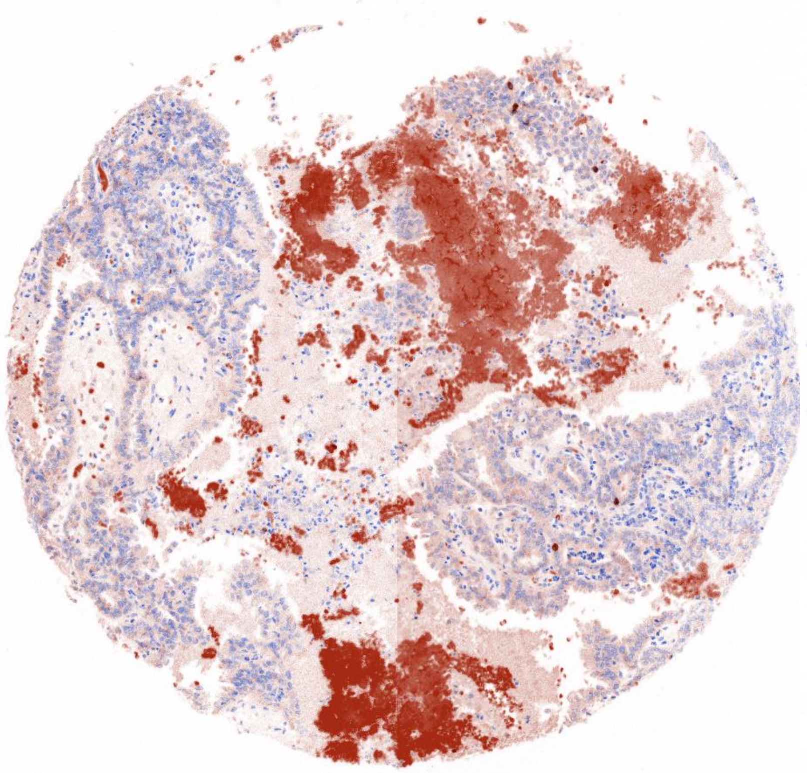

Grade 0 example 1

(High background staining)

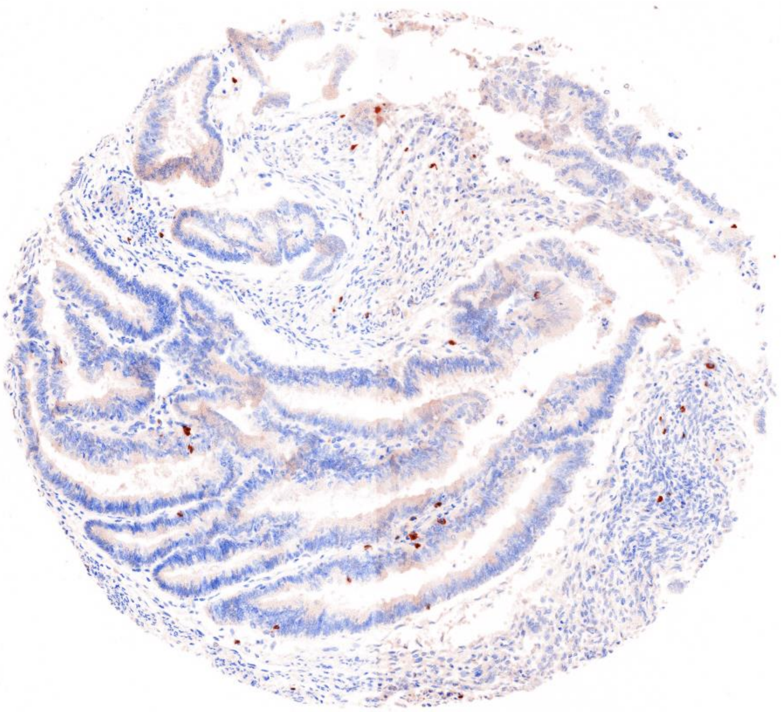

Grade 0 example 2

CD8 GRADE 1 & 2 (+/++)

Intra-tumoral lymphocytes seen in most fields, without reaching high infiltration densities.

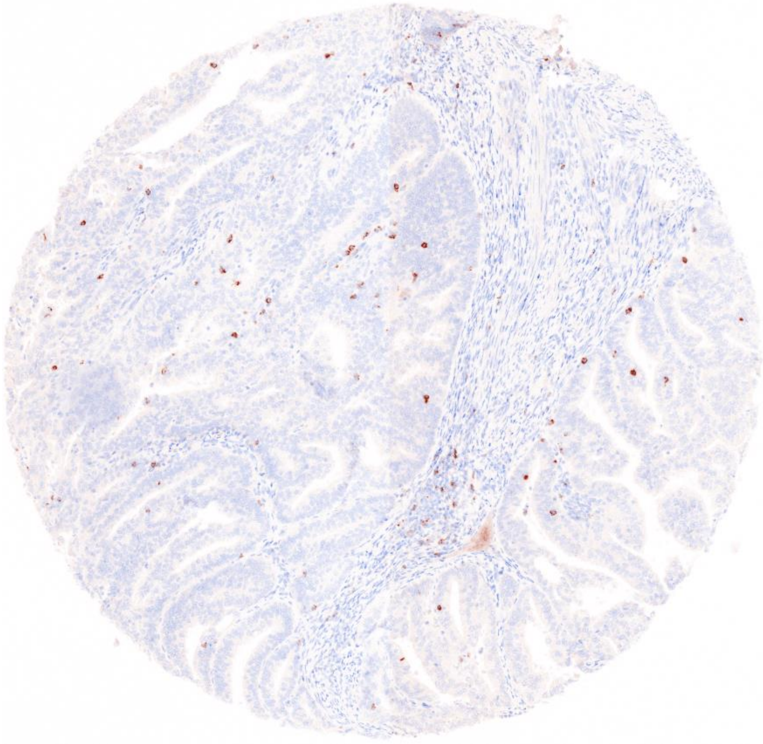

Grade 1/2 example 1

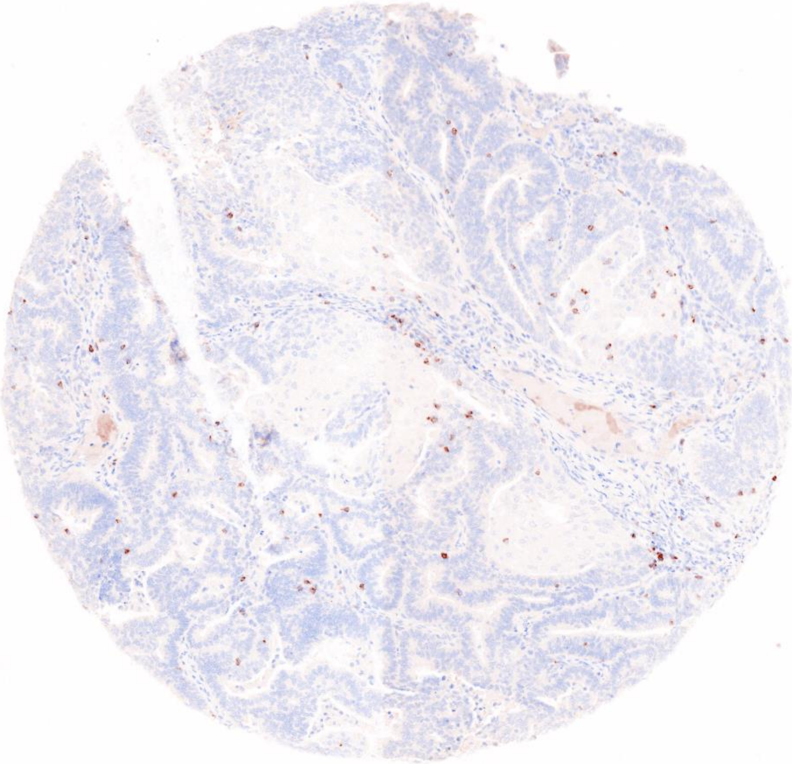

Grade 1/2 example 2

CD8 GRADE 3 (+++)

Frequently encountered intra-tumoral lymphocytes .

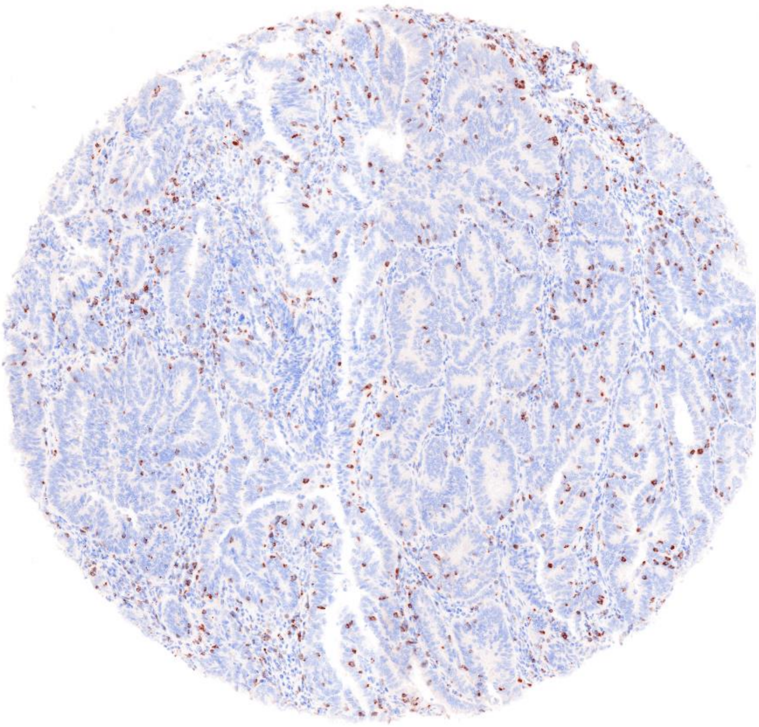

GRADE 3 Example 1

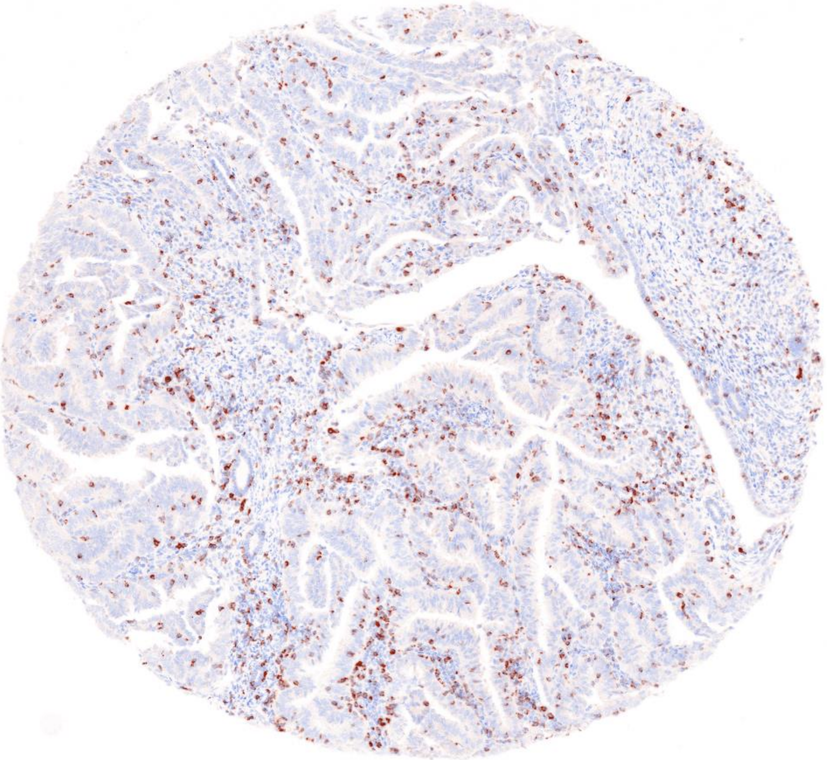

GRADE 3 Example 2

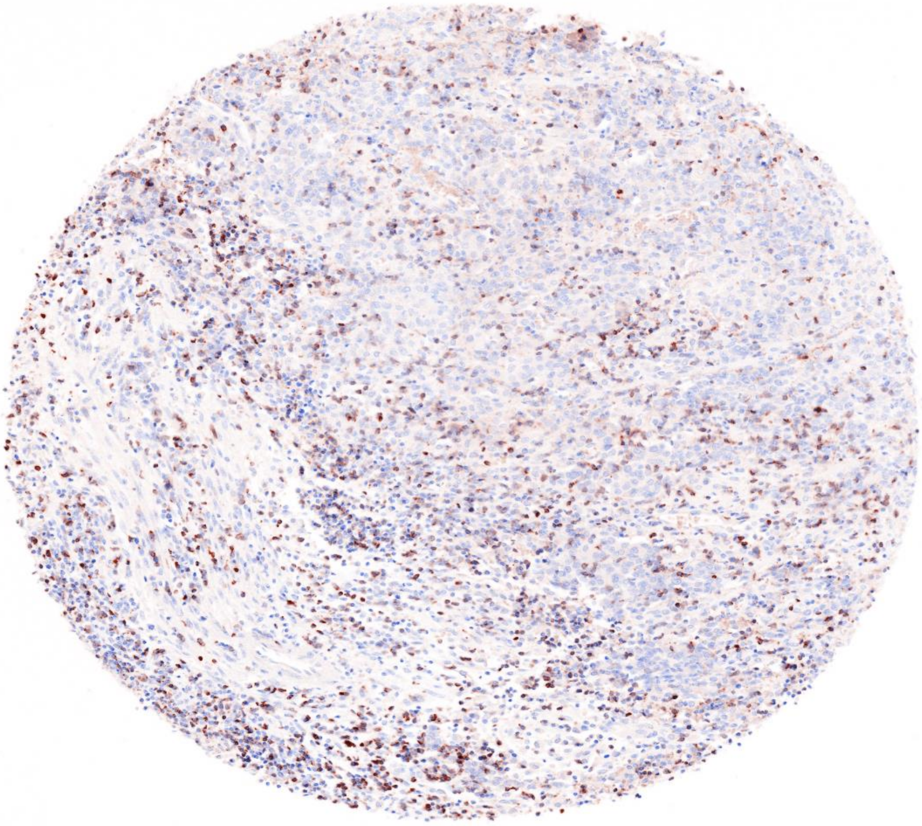

GRADE 3 Example 3

CD68

CD68 GRADE 0 (0+)

Absent or very infrequent INTRA-TUMOR macrophages.

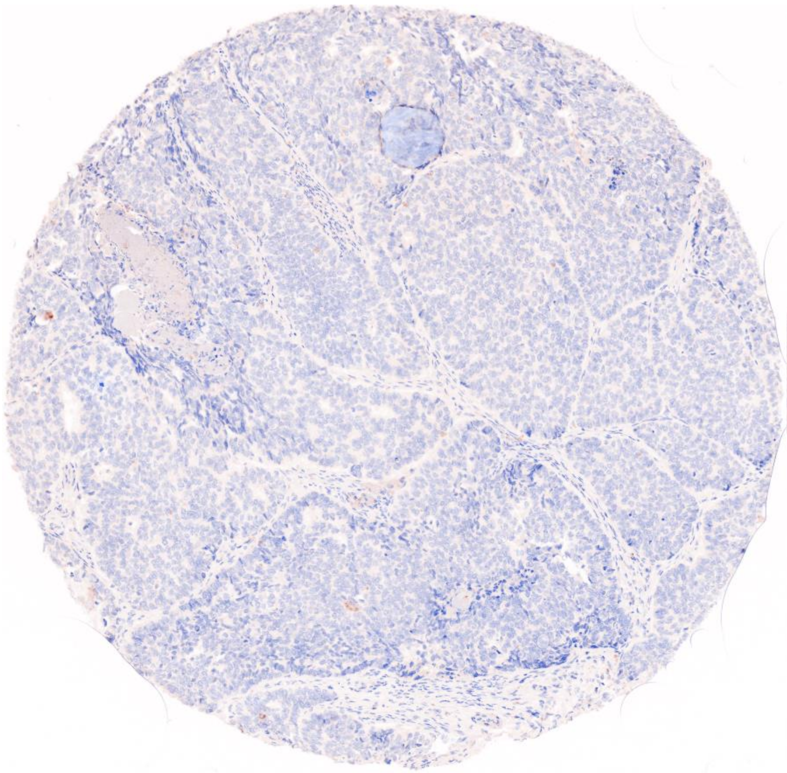

Grade 0 example 1

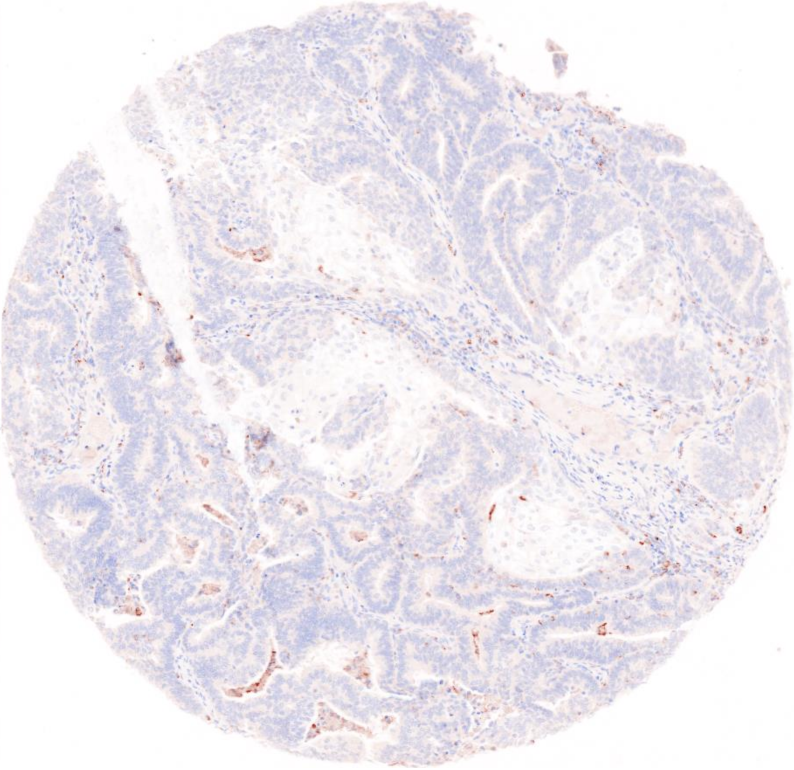

Grade 0 example 2

CD68 GRADE 1 & 2 (+/++)

Frequent INTRA-TUMOR macrophages. Stain may be faint, consider any positive cell when inferring marker density.

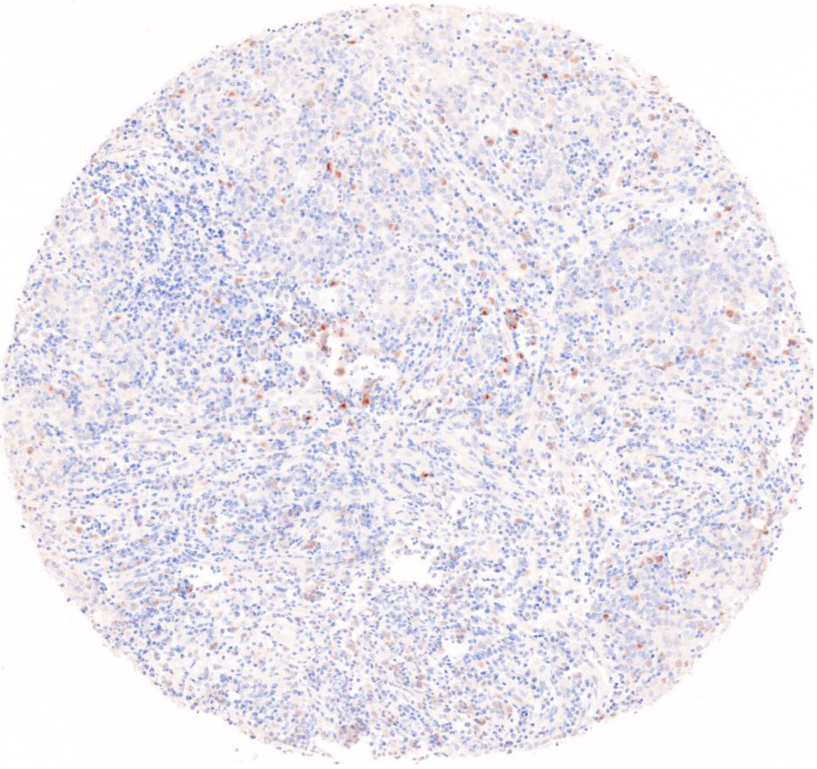

Grade 1/2 example 1

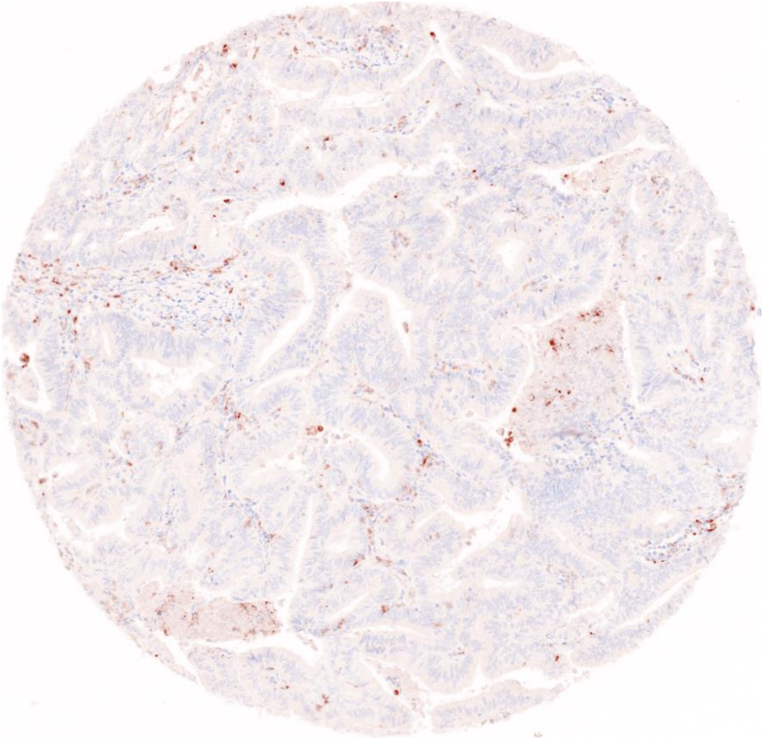

Grade 1/2 example 2

CD68 GRADE 3 (+++)

High density of macrophages. Intra-luminal CD68+ cells should be taken into account.

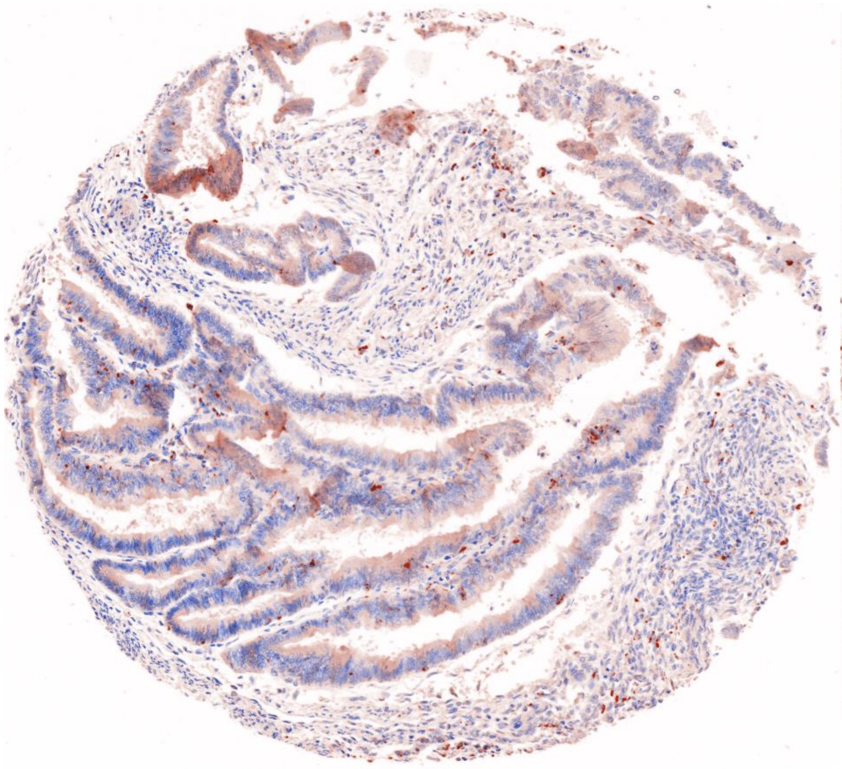

Grade 3 example 1

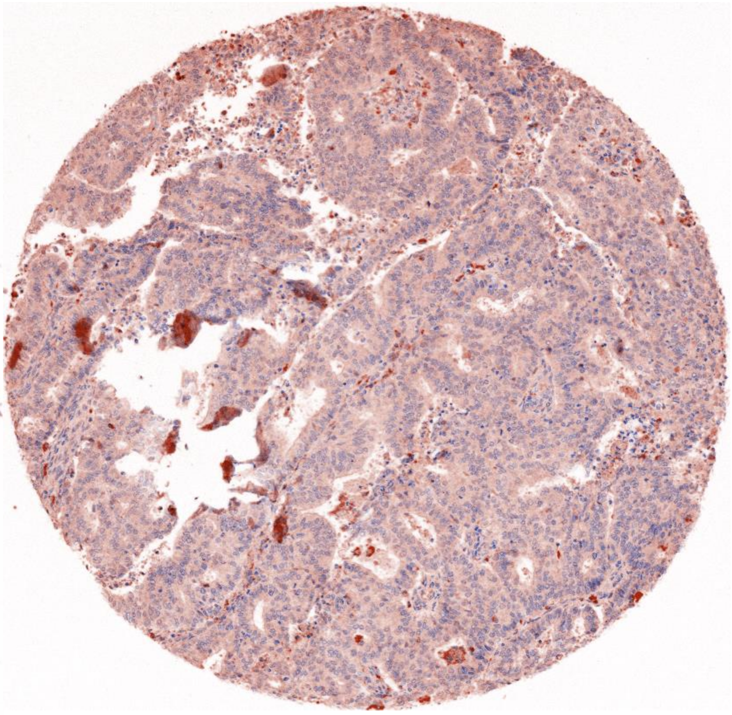

Grade 3 example 2. Account for intraluminal CD68+cells

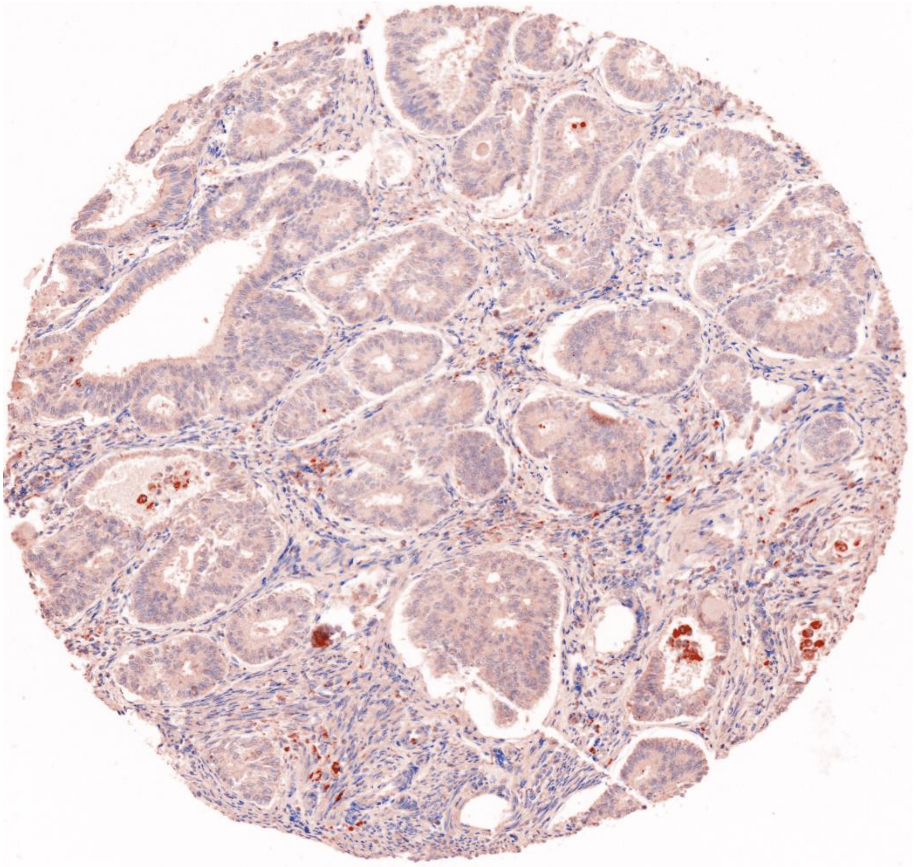

Grade 3 example 3. Account for intraluminal CD68+cells

FOXP3

FOXP3 GRADE 0 (0+)

Complete absence of FOXP3 stained nuclei in the tumor compartment.

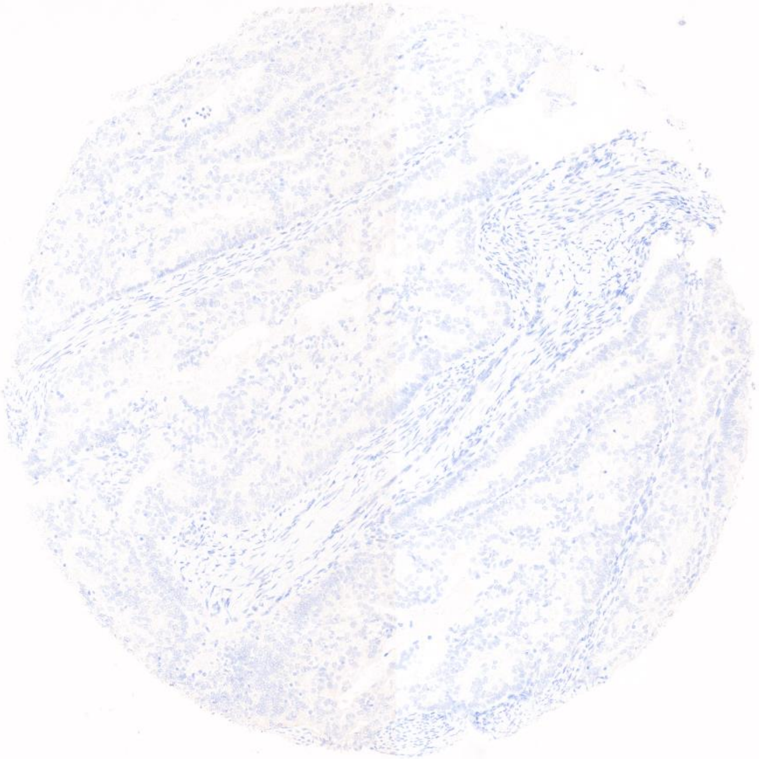

Grade 0 example 1

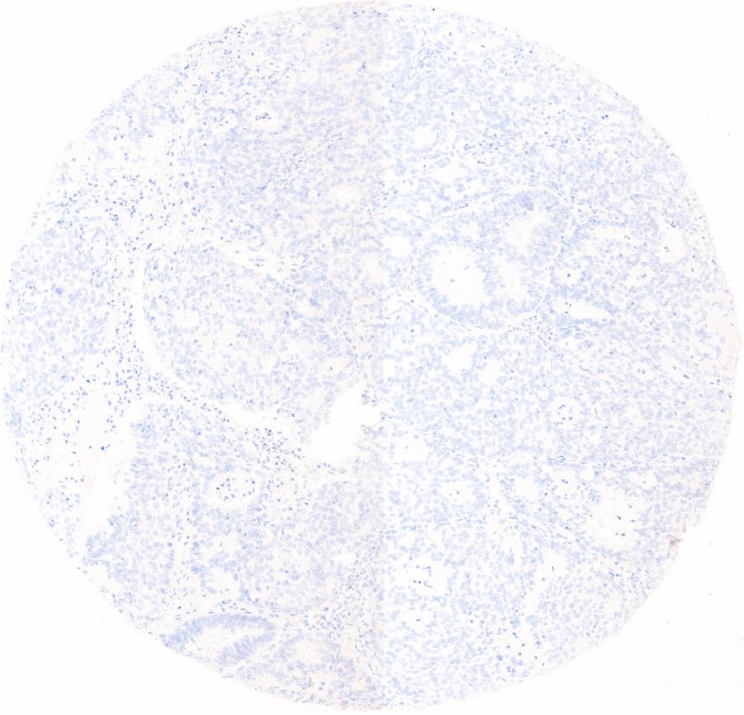

Grade 0 example 2

FOXP3 GRADE 1 (+)

Presence of ANY amount of FoxP3 stained nuclei in the tumor (faint nuclear staining should also be scored).

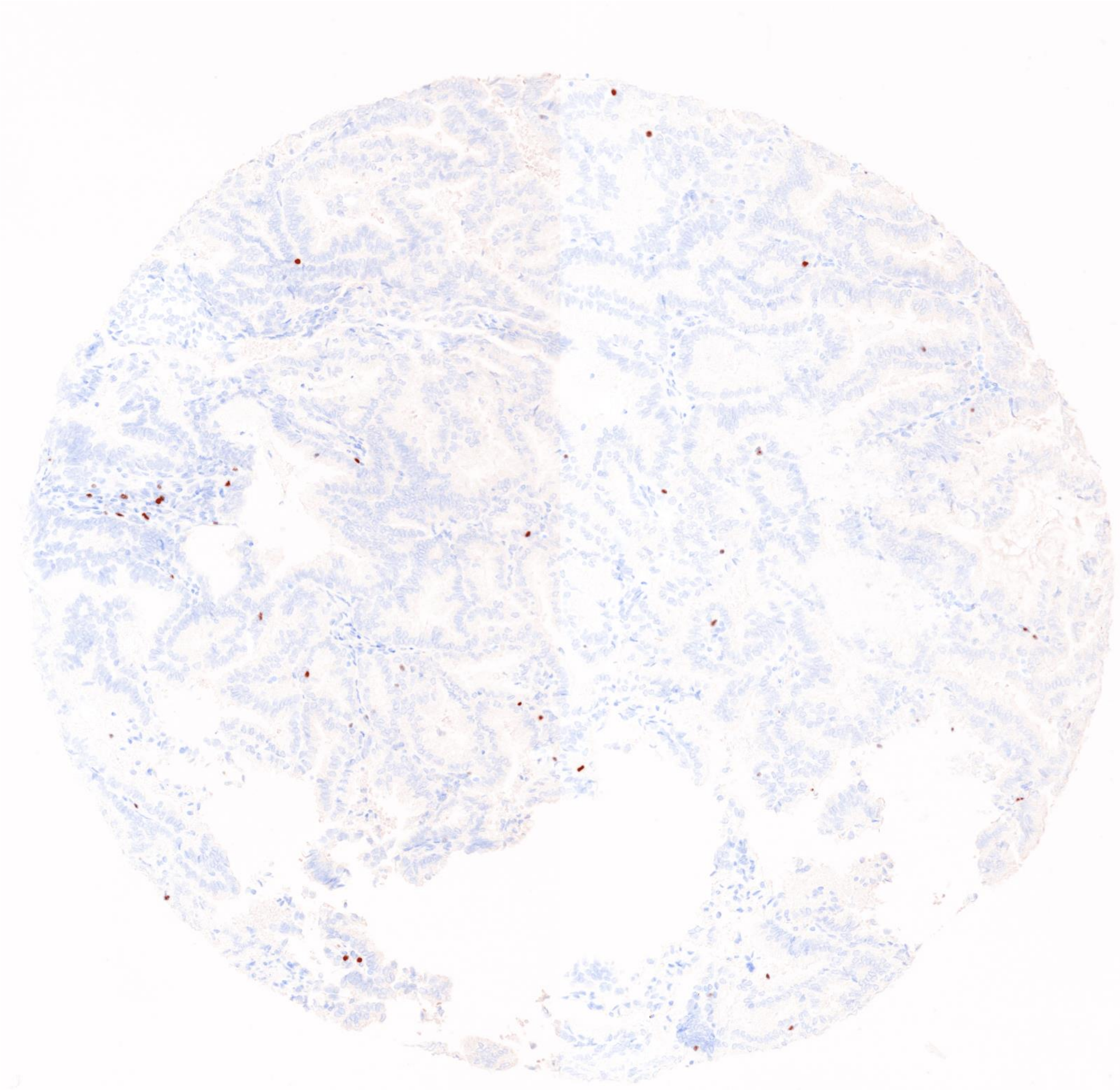

Grade 1 example 1

PDL1

PDL1 GRADE 0 (0+)

Tumor cells lack PDL1 expression. Avoid scoring intratumoral immune cells.

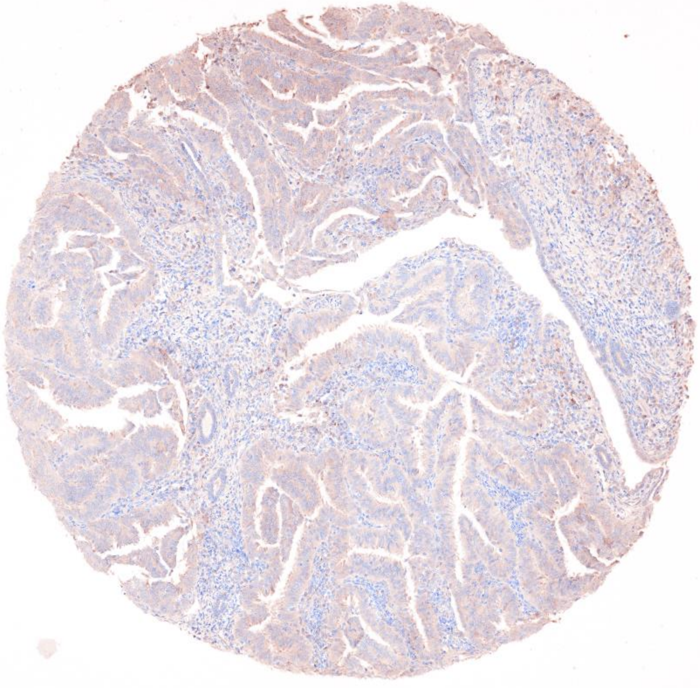

Grade 0 example 1

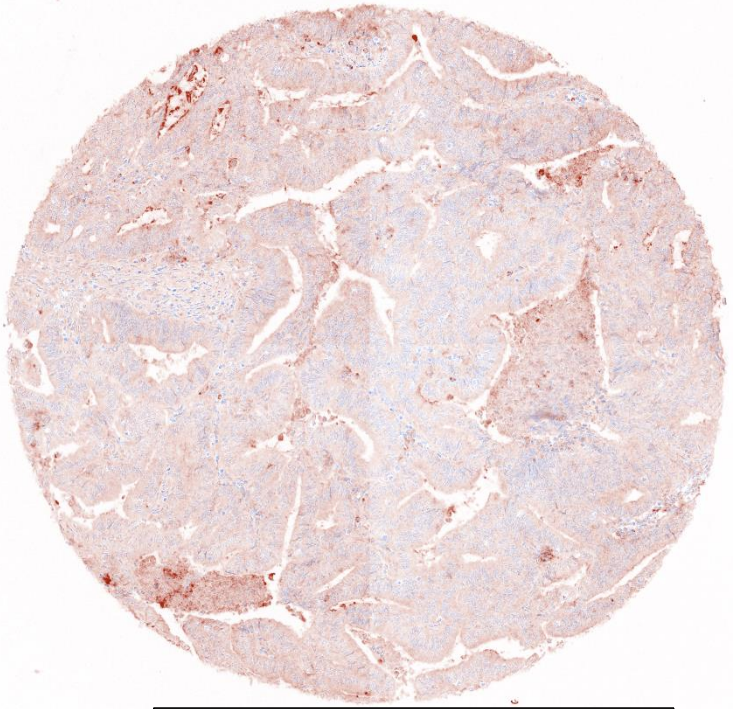

Grade 0 example 2. Avoid scoring intraluminal immune cells

PDL1 GRADE 1 (+)

Presence of ANY amount of PDL1 stained tumor cells (faint incomplete staining should also be scored). Staining may be membranous or cytoplasmic.

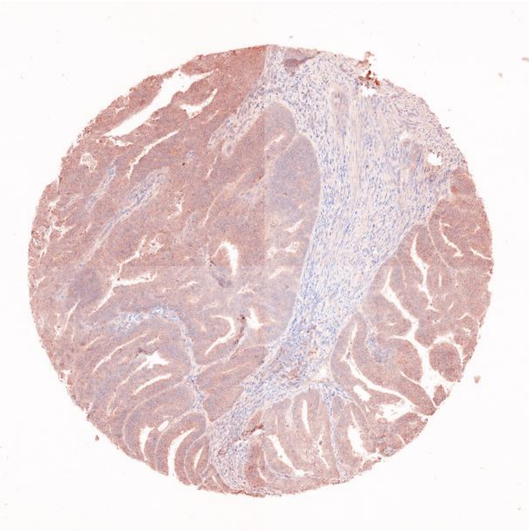

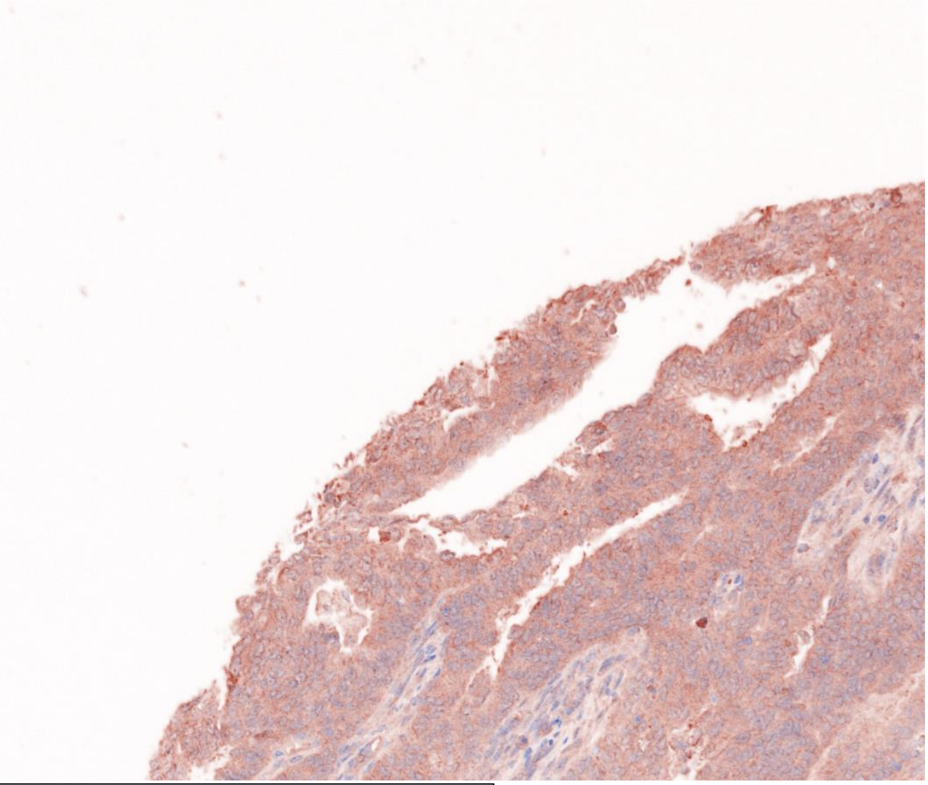

Grade 1 example 1. Note focal expression of PDL1

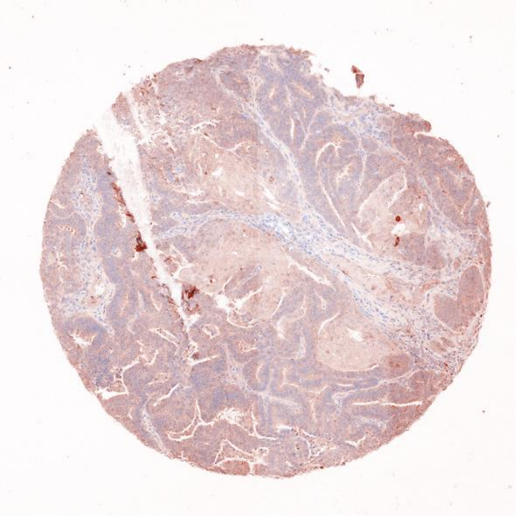

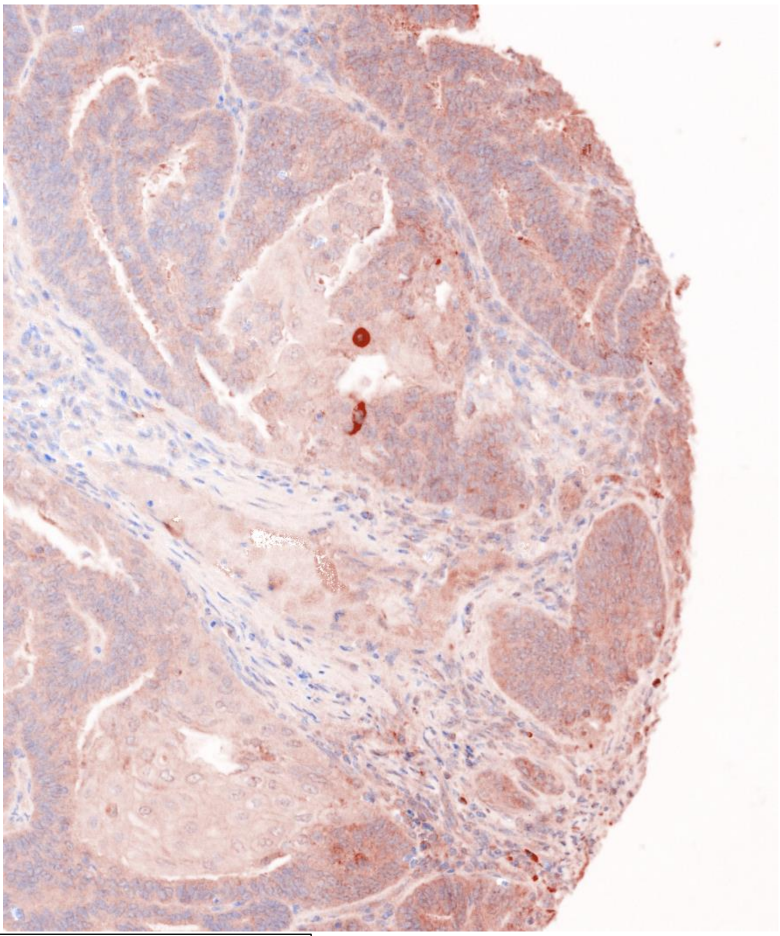

Grade 1 example 2. Note focal expression of PDL1
